# Is university attendance associated with differences in health service use for a mental health problem in emerging adulthood? Evidence from the ALSPAC population-based cohort

**DOI:** 10.1007/s00127-025-02922-3

**Published:** 2025-05-19

**Authors:** Tom G. Osborn, Rob Saunders, Peter Fonagy

**Affiliations:** 1 CORE Data Lab, Research Department of Clinical, Educational and Health Psychology, 1-19 Torrington Place, UCL, London, WC1E 7HB England; 2 Division of Psychology and Language Sciences, 26 Bedford Way, London, WC1H 0AP England

**Keywords:** Emerging adulthood, Health service use, University, Higher education, ALSPAC

## Abstract

**Purpose:**

It is unclear whether attending university is associated with health service use for mental health problems in emerging adulthood. As this can be a marker of the onset of mental disorders, we aimed to investigate whether attending university was associated with health service use for a mental health problem by age 24.

**Methods:**

We used data from the Avon Longitudinal Study of Parents and Children (ALSPAC). The analytic sample comprised of 2,649 individuals with data on university attendance reported approximately between ages 25 and 26, and health service use for a mental health problem reported around age 24. Logistic regression models were used to investigate the association between university attendance and health service use, employing confounder adjustment, multiple imputation and propensity score matching to assess the robustness of associations. The study was reported using STROBE guidelines.

**Results:**

University attendees were less likely to report having used services for mental health problems by 24 years compared to non-university attendees (6.5% vs. 11.4%, odds ratio (OR) = 0.54[95%CI = 0.40;0.72], *p* < 0.001). This association was robust in the fully adjusted model (aOR = 0.38[95%CI = 0.15;0.94], *p* = 0.04), propensity score matching and multiple imputation. There was evidence of a differential association among those who were and were not heterosexual and according to maternal education level.

**Conclusions:**

Our findings suggest individuals who attend university are less likely to use a health service for a mental health problem. Further longitudinal research is needed to investigate potential explanations for these differences.

**Pre-registration:**

A study protocol was submitted to the ALSPAC team.

**Supplementary Information:**

The online version contains supplementary material available at 10.1007/s00127-025-02922-3.

## Introduction

Young adulthood is a crucial stage for mental health, as around 75% of lifetime mental disorders start before age 25 [1]. The mental well-being of young adults is increasingly worrisome, evidenced by rising occurrences of symptoms associated with mental disorders in the past two decades [2, 3]. Concurrently, more young adults are acknowledging mental health problems and are actively seeking assistance, with health services experiencing heightened demand [4], especially within university environments [5]. Recognizing these developments during this critical life phase, enhancing the mental health of young adults has become a key objective of public health and policy initiatives in numerous high-income countries, including the United Kingdom (UK) [6].

University students constitute a significant portion of young adults, with their mental health becoming a growing area of concern [5, 7]. This group has expanded in the past two decades, encompassing a broader spectrum of backgrounds and needs [7, 8]. Aspects of university life may elevate mental health risks for certain young adults by leaving one’s social support network, from new financial and academic pressures, and heightened engagement in risk behaviours like substance abuse [9, 10]. Attending university might also offer some protection for mental health, through accruing occupational advantages [11], learning effective psychological coping strategies [12], and having opportunities to develop social supports [10]. Therefore, comparative studies of mental health requirements between university students and non-students are essential to ensure that public health strategies are fair and to comprehend any potential mental health risks related to university attendance.

There are a small number of studies which investigated differences between students and non-students [13–15]. For instance, an analysis of the 2007–2014 UK National Psychiatric Morbidity Surveys found no difference in the prevalence of symptoms of Common Mental Disorders (CMD) [14]. An analysis of the Understanding Society Cohort found that students aged between 17 and 24 had lower average distress and case-level distress based on the General Health Questionnaire (GHQ) [13]. A third study investigated symptom trajectories over time using the Longitudinal Survey of Young People in England (LSYPE). This found symptoms of distress increased between ages 18–19 among students when compared to non-students, but the difference disappeared by age 25 [15]. These findings suggest the transition into the university environment could be associated with increased mental health risk for some students, although the extent to which any increases in symptoms translate into clinically significant mental health problems is less clear.

While extant epidemiological studies have employed thoroughly validated methods for assessing mental health symptoms, they have not explored variations in health service utilization due to health problems. Investigating these differences in health service utilisation is crucial, as symptom assessments may not reflect the need for treatment of mental disorders, whereas patterns of help-seeking and service usage could signal the onset of such disorders [16, 17]. Differential patterns of service utilisation and help-seeking might also signal barriers to access. Several qualitative studies have delineated barriers related to the students’ help-seeking, such as a university social environment perceived as valuing self-reliance, and significant selectivity of health services that might explain any differential patterns of service utilisation [18–22]. Identifying disparities in service use between students and non-students is key to pinpointing those at increased risk of adverse long-term consequences and for devising public health strategies that prevent exacerbating social inequalities among these groups.

To date, only cross-sectional studies have examined variances in health service utilization in students, indicating differences by student groups. An international survey revealed that students aged 18–22 report a higher incidence of minimally adequate treatment compared to their non-student counterparts [23]. While this indicates students may be more likely to engage with necessary intervention once reaching services, it does not indicate differences in help-seeking, which are important to understand mental disorder onset. Another study found students with lower parental education were less likely to report service use, while being female and heterosexual were more likely to seek help in the previous 12 months [24]. While helpful for identifying differential within-group differences in health service use, this study and recent UK-based research have not compared student and non-student populations [25, 26]. Moreover, a common limitation of cross-sectional designs is their lack of representativeness, often due to recruitment challenges. Therefore, to bridge these gaps, our study employs data from a population-based cohort. It aimed to determine if there are differences in health service usage for mental health problems between individuals who have, and those who have not, attended university by age 24.

## Method

### Study design and participants

We used data from the Avon Longitudinal Study of Parents and Children (ALSPAC) [27–29]. ALSPAC is a longitudinal general population birth cohort study of children, who are now adults, and their parents. All children in the study were born in the former county of Avon, an area centred around the city of Bristol. Original recruitment for the cohort took place over the course of 22 months, targeting mothers with expected delivery dates between April 1, 1991, and December 31, 1992. As of February 2019, out of 15,447 eligible pregnancies, 14,901 children were alive at one year of age and could be included in the analytic sample. The protocol submitted to the ALSPAC group to gain access to the data is presented in the Online resources, alongside deviations from this protocol (see Online resource 1). The study is reported in accordance with the STROBE guidelines for observational studies (see Online resource 2).

Study data were collected and managed using REDCap electronic data capture tools hosted at the University of Bristol. REDCap (Research Electronic Data Capture) is a secure, web-based software platform designed to support data capture for research studies [30]. The study website contains details of all the data that is available through a fully searchable data dictionary and variable search tool: http://www.bristol.ac.uk/alspac/researchers/our-data/.

Participants were selected for the current analysis if they:

1. Were alive at one year.
2. Had complete data on at least one of four items indicating whether the individual had attended university, reported at approximately ages 25 and 26.
3. Had complete data for health service use reported at approximately age 24.

### Procedures and measures

Full details on the data used to derive the exposure, outcome and confounders are in Table 1. The main exposure variable of our study was university attendance, distinguished by individuals who reported attending university at least once versus those who did not. We created a binary variable– attended or not attended university– derived from four specific criteria from life events and education questionnaires (refer to Table 1).

**Table 1:** Variables used in analyses

Concept	Indicator	Time point	Additional information
University attendance	Binary: attended / not attended university	~Age 25–26	Coded as non-attendees as “0” and attendees as “1”. Variable created from four items in the ALSPAC life events and education questionnaires: 1. Year entered university or did not attend at all, reported at approximately age 25. 2. Year left university or did not attend at all, reported at approximately age 25 3. Graduated in the previous 12 months at approximately age 25. 4. Graduated in the previous 12 months at approximately age 26. Individuals were coded as ‘non-attendee’ if they reported not attending. Individuals were coded as ‘attendee’ if they reported a university start and end date or graduating.
Any health Service Use	Binary: used / did not use	~Age 24	Created from the PLIKS questionnaire [31, 32]. Utilisation coded as “1”, other “0” for did not use.
GP service use	Binary: used / did not use	~Age 24	Created from the PLIKS questionnaire [31, 32]. Utilisation coded as “1”, other “0” for did not use.
Counsellor service use	Binary: used / did not use	~Age 24	Created from the PLIKS questionnaire [31, 32]. Utilisation coded as “1”, other “0” for did not use.
Mental Health Service Use	Binary: used / did not use	~Age 24	Created from the PLIKS questionnaire [31, 32]. Utilisation coded as “1”, other “0” for did not use.
Medication use	Binary: used / did not use	~Age 24	Created from the PLIKS questionnaire [31, 32]. Utilisation coded as “1”, other “0” for did not use.
Depressive symptoms	Continuous: Short Mood and Feelings Questionnaire total score (sMFQ)	~Age 17 and 21	sMFQ is a 12-item questionnaire used as a screening tool for depression in children and young people aged 6 to 19 (Turner et al., 2014). Items are descriptions where respondents are asked whether these descriptions are “not true”, “sometimes true”, or “true”, scored as 0, 1, and 2 respectively. The sMFQ items are summed, leading to the total scores of between 0 to 26 with higher scores indicating great symptoms of depression. Scoring 12 or higher may indicated the presences of depression.
Probable depression	Binary: likely depression or not	~Age 17 and 21	For individuals with short mood and feeling questionnaire scores of 12 or more were coded as ‘1’ for ‘probable depression’, otherwise coded as ‘0’ for ‘below threshold’ [33].
Sex assigned at birth	Binary: male or female	Birth	Individuals recorded as ‘0’ for male, and ‘1’ for female. SAGAR guidance was adhered to when reporting on sex [34].
Ethnicity	Binary: white or non-white	Birth	Individuals recorded as ‘0’ for white, and ‘1’ for minoritized ethnicity.
Maternal highest education	Binary: compulsory or non-compulsory	Gestation	Original variable has four response options “Certificate of Secondary Education (CSE)”, “Vocational”, “O Level”, “A Level”, or “Degree”. Binary variable was created where any individuals reporting either CSE, Vocational or O Level was coded as ‘0’ or ‘Compulsory’, A level or Degree was coded as ‘1’ or ‘non-compulsory’, following.
Family composition	Binary: married/ cohabiting, or not	Birth	Created a new variable from ‘partner status’ and ‘marital status’ variables. Individuals were coded as ‘0’ or ‘Married/cohabiting’ if individuals reported they were married, partnered or cohabiting, and as ‘1’ if they were not.
Neighbourhood level deprivation	Ordinal: Index of Multiple Deprivation Quintiles	Gestation	Coded as ‘0’ for least deprived, ‘1’ for 2nd least, ‘2’ for 3rd least, ‘3’ for 4th least, ‘4’ for most deprived.
Adverse Childhood Experiences (ACE)	Ordinal: zero ACEs, one ACE, or 2 or more ACEs.	Between 0–16.	Created a new variable from variables which recorded if an individual had experienced ‘physical abuse’ or ‘sexual abuse’ or ‘emotional abuse’ or ‘neglect’ or ‘bullying’ or ‘domestic violence’ [35]. If an individual had not reported any ACEs then they were coded as ‘0’ for zero ACEs, ‘1’ for one ACE, or ‘2’ for two or more ACEs’.
Autistic traits	Binary: below threshold or above threshold.	Age 16	Social and Communications Difficulties Checklist (SCDC) [36]. Individuals could answer as ‘Not True’ coded as ‘0’, ‘Sometimes True’ coded as ‘1’, or ‘True’ coded as ‘1’. First variable created was a total score by summing the scores from the items of the SCDC. A binary variable was created using the total score variable, where individuals were coded as ‘0’ or below threshold if their total score 8 or below and coded as ‘1’ or above threshold if their total score was 9 or above.
Disability status	Binary: disability or no disability	Age 18	Individuals were asked if they consider themselves to have a long-standing disability, illness or infirmity. If they did, they were coded as ‘0’, otherwise ‘1’.
Carer status	Binary: carer or not	Age 22	Individuals were asked if they were currently a full-time or part-time carer. Individuals were coded as ‘0’ if a carer, or ‘1’ if not a carer. We assumed carer status recorded at age 22 was reflective of status prior to attending university.
Sexual orientation	Binary: heterosexual or not heterosexual	Age 23	A binary variable was created out of sexual orientation variable which had eight response options. Participants were coded as ‘0’ if they were reported they were 100% heterosexual, otherwise they were coded as ‘1’ if not heterosexual. We assumed sexual orientation recorded at age 23 was reflective of sexual orientation prior to attending university.

The primary outcomes of interest were the use of health services for a mental health problem by age 24. We employed items from the Psychotic-Like Symptoms (PLIKS) questionnaire, completed around age 24 [31, 32]. This questionnaire includes distinct queries about whether the participant has consulted a general practitioner, counsellor, mental health service, or has been prescribed medication for hallucinations/delusions or any other mental health problem. Additionally, all participants were asked if they had not sought any assistance for their hallucinations/delusions or other reported mental health problems around age 24.

The primary outcome was defined as any use of health services, determined by whether an individual reported using any of the services mentioned in the PLIKS questionnaire for hallucinations/delusions or any mental health problem at approximately age 24. We also considered four secondary outcomes based on reports of using (1) a general practitioner, (2) a counsellor, (3) a mental health service, or (4) medication. For all these outcomes, the reference category used was no service use.

To evaluate the association between university attendance and health service use, we considered several potential confounding factors. An established framework of health service use was adopted to guide the inclusion of these variables into the models [37]. These included predisposing factors (i.e., demographic and social conditions influencing an individual’s decision to use services) of sex assigned at birth, ethnicity recorded at birth, sexual orientation reported at age 23, adverse childhood experiences (ACE) between the ages of 0–16 based on self-reports of physical abuse, sexual abuse, emotional abuse, neglect, bullying or domestic violence [35], and family composition; enabling factors (i.e., economic circumstances that facilitate service utilisation) of maternal education level, neighbourhood deprivation level at gestation, and carer status at age 22; and need factors (i.e., perceived or actual health needs) included autistic traits at age 16, determined by a Social and Communication Disorders Checklist (SCDC) total score above 8 [36, 38]) and self-reported disability status at age 18. We also included probable depression at age 17, identified by a Short Mood and Feelings Questionnaire (sMFQ) score of 12 or above [33]. Depression scores were included as individuals with higher depressive symptoms may be less likely to attend university [13] and more likely to be in contact with health services [23]. Moreover, the sMFQ measured at age 17 may not fully reflect an individual’s range of mental health symptoms while at university. Therefore, any continued association could indicate other mental health problems not accounted for in this analysis.

### Statistical analysis

To explore the association between university attendance and health service use for a mental health problem by age 24, we employed logistic regression models. Initially, we conducted a series of univariate analyses to examine the relationship between each confounder, the exposure, and the outcome. Recognising the established inequalities in university attendance and health service use by demographic characteristics, we planned to include variables sex assigned at birth, ethnicity, sexual orientation, maternal education, and disability status a priori. Other confounders were added to subsequent models only if they reached a significance threshold of *p* < 0.05 in univariate analysis.

Next, we modelled the relationship between university attendance and health service use for a mental health problem, controlling for confounders in a stepwise manner across four models. The first model was unadjusted, focusing solely on the exposure and outcome variable. Model 2 incorporated predisposing factors for service use, namely sex assigned at birth, sexual orientation, and ethnicity. Model 3 included family composition. Model 4 was adjusted for enabling factors; maternal highest education level and Index of Multiple Deprivation (IMD) score at gestation. In model 5, need variables; probable depression, disability status, and autistic traits were included.

Interaction terms were incorporated into a logistic regression model to assess the differential impact of university attendance based on individual factors such as sex assigned at birth, ethnicity, sexual orientation, and maternal highest education level. We also included confounders that consistently showed an association with the primary outcome in model 5 in interaction models.

To assess the robustness of our findings in the complete case analysis, we employed propensity score matching [39]. To make the most of the available sample, only confounders that demonstrated an association in multivariate logistic regression models or were predictors of university attendance were used in the matching algorithm. This included sex assigned at birth, sexual orientation, maternal highest education level, probable depression, and autistic traits. We set the caliper at 0.02 standard deviations of the propensity score [40] and matched each case to its nearest neighbour to ensure optimal pairings in all scenarios [39, 41]. In the analysis, each case was weighted according to the number of matches.

To further verify the robustness of our findings, all analyses, except the matched model, were repeated after addressing missing data. We used Multiple Imputation by Chained Equations (MICE) to impute missing data for all variables in the analytic sample [42]. With no variable exceeding 50% missing observations, fifty datasets were generated [41, 43]. Analyses were conducted on each dataset, and the results were then aggregated across these datasets [42].

A sensitivity analysis examined the association between university attendance and health service use for a mental health problem only in individuals with probable depression at age 17.

### Ethical considerations

Ethical approval for the study was obtained from the ALSPAC Ethics and Law Committee and the Local Research Ethics Committees. Informed consent for the use of data collected via questionnaires and clinics was obtained from participants following the recommendations of the ALSPAC Ethics and Law Committee at the time.

## Results

### Participants

**Fig. 1:**
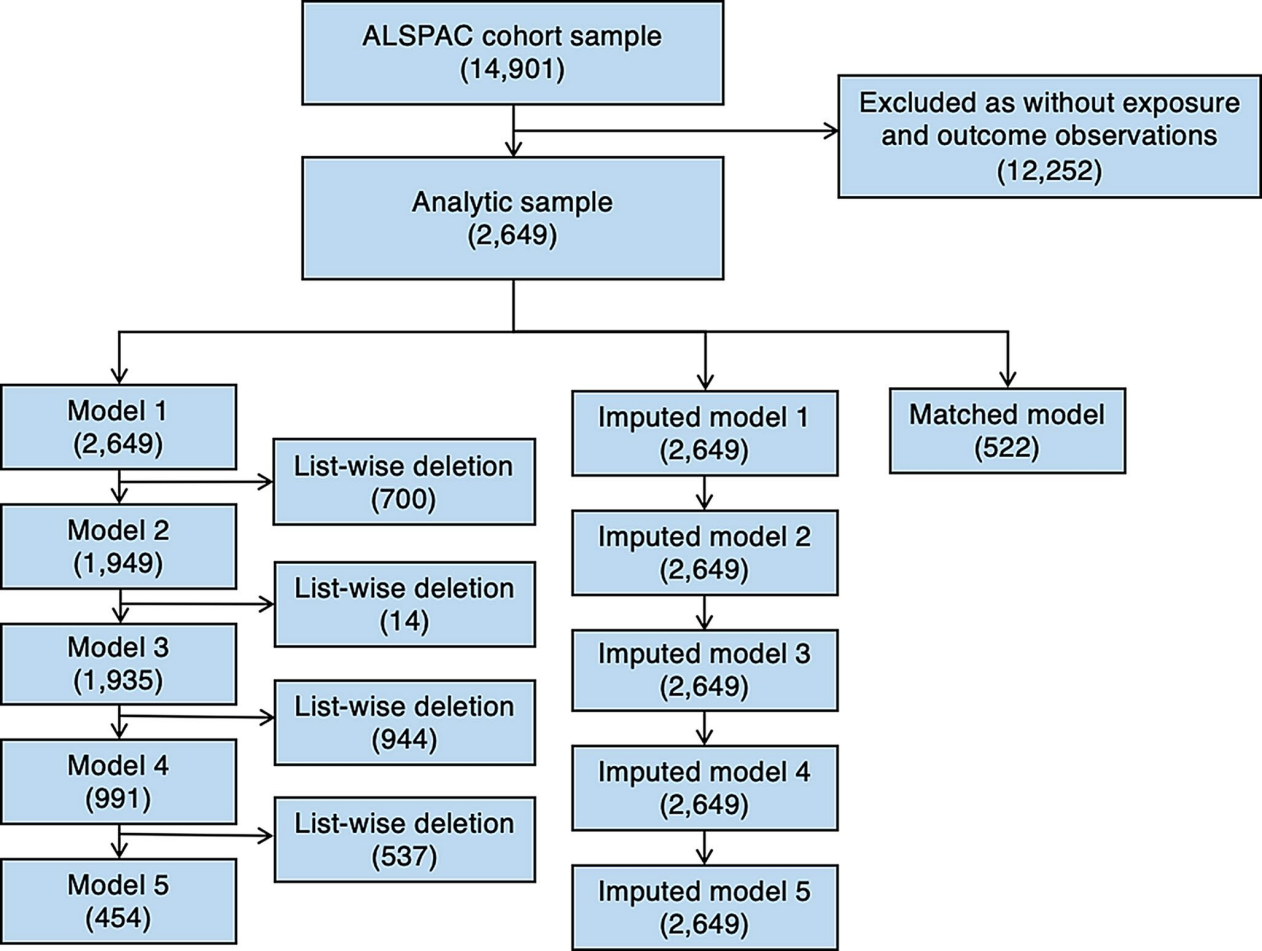
Flow diagram of the analytic process

Of the 14,901 participants in the ALSPAC dataset, 2,649 (17.8%) had complete data for both university attendance and health service use at age 24, forming the analytic sample (refer to Fig. 1). Differences in the analytic and excluded samples were observed across all included variables except disability status (χ2 = 0.55; *p* = 0.46) (see Online Resource 3).

In the analytic sample, all variables exhibited some degree of missing data, with the Index of Multiple Deprivation (IMD) score showing the highest proportion of missing data (48.3%[*n* = 1,278]) and sex assigned at birth having the lowest (0.1%[*n* = 2]). Variations were noted in the distribution of variables between those who did and did not attend university; based on sexual orientation (*p* = 0.04), family composition at gestation (*p* < 0.001), maternal highest education (*p* < 0.001), IMD quintiles (*p* < 0.001), probable depression (*p* < 0.001), above threshold autistic traits (*p* < 0.001), carer status (*p* = 0.002), using any health service (*p* < 0.001), using general practice (*p* < 0.001), using counselling (*p* = 0.004), using mental health services (*p* = 0.05), and using medication (*p* < 0.001) for a mental health problem (refer to Table 2).

**Table 2:** Differences across included variables in attendees and non-attendees

	Attendees		Non-attendees			
Variable	N	%	N	%	χ^2^	p-value
Sex assigned at birth						
Female	1,276	67	512	69		
Male	626	33	233	31	0.7	0.42
Ethnicity						
White	1,660	96	626	97		
Not white	61	4	23	3	0.0	0.99
Sexual Orientation						
Heterosexual	1,213	77	463	80		
Not heterosexual	378	23	113	20	4.1	0.04
Family composition at gestation						
Married or co-habiting	1,520	87	536	80		
Not married or co-habiting	227	13	134	20	18.7	< 0.001
Maternal highest education						
Compulsory only	1,154	70	516	92		
Non-compulsory	487	30	42	8	111.8	< 0.001
IMD^a^ quintile at gestation						
Least deprived	367	37	94	24		
2nd	185	19	65	16		
3rd	169	17	64	17		
4th	138	14	76	20		
Most deprived	122	12	93	24	44.5	< 0.001
Disability at age 18						
Yes	185	15	66	17		
No	1049	85	331	83	0.62	0.43
sMFQ^b^ score at age 17						
< 12	1,270	87	365	76		
≥ 12	190	13	116	24	33.59	< 0.001
Autistic traits at age 16						
Below threshold	1,352	94	424	89		
Above threshold	81	6	52	11	15.3	< 0.001
Adverse childhood experiences between age 0–16						
0	1,383	53	711	52		
1	765	29	377	28		
≥ 2	477	18	282	21	3.70	0.16
Carer status						
Carer	39	2	15	5		
Non-carer	1,567	98	336	95	9.80	0.002
Any health service use for a mental health problem by age 24						
Yes	123	6	85	11		
No	1,781	94	660	89	18.13	< 0.001
General Practice use for a mental health problem by age 24						
Yes	104	5	78	10		
No	1,800	95	667	90	20.99	< 0.001
Counselling use for a mental health problem by age 24						
Yes	75	4	49	7		
No	1,829	96	696	93	8.35	0.004
Mental health service use for a mental health problem by age 24						
Yes	60	3	35	5		
No	1,844	97	710	95	3.71	0.05
Medication use for a mental health for a mental health problem by age 24						
Yes	68	5	52	7		
No	1,836	95	693	93	14.38	< 0.001

^a^Index of Multiple Deprivation, ^b^Short Mood and Feelings Questionnaire

In the complete case analysis sample of 2,649 participants, 71.9% (*n* = 1,904) reported attending university. The majority of these participants were female (67.5%,*n* = 1,788/2,647) and white (96.5%,*n* = 2,286/2,369). The period of university attendance ranged from 2008 to 2018 (M = 2010;SD = 1.8 years) and leaving years between 2009 and 2019 (M = 2014;SD = 1.60 years). A smaller percentage of university attendees reported using a health service for a mental health problem (6.5%[95%CI:5.4;7.7]) compared to those who did not attend university (11.4%[95%CI:9.3;13.9]). Additionally, a higher proportion of non-attendees (25.8%[95%CI:22.7;29.2]) than attendees (13.4%[95%CI:11.9;15.1%]) had a score above 12 on the sMFQ at age 17 (refer to Online Resource 4**).**

### Health service use for a mental health problem and university attendance

The primary multivariate analyses are presented in detail in Table 3, with comprehensive information on univariate and multivariate analyses available in Online Resources 5–10. Unadjusted Model 1 indicated that university attendees had a 46% lower odds of using health services for mental health problems (OR = 0.54[95%CI:0.40–0.72], *p* < 0.001). In Model 2, the odds of health service use before age 24 were 56% lower for university attendees (aOR = 0.44[95%CI:0.30;0.63], *p* < 0.001). Model 3 showed a 53% reduction in the odds of health service use before age 24 for university attendees (aOR = 0.47[95%CI:0.32;0.68], *p* < 0.001). In Model 4, the odds were 55% lower (aOR = 0.45[95%CI:0.25;0.79], *p* = 0.006), and in Model 5, they were 63% lower (aOR:0.37[95%CI: 0.14;0.93]*p* = 0.04). Overall, there was a trend of lower odds of health service use across all secondary outcomes in university attendees compared to non-attendees, but no statistical evidence for the mental health service outcome.

**Table 3:** Multivariate analyses modelling the relationship between university attendance and health service use by age 24

Model (*n*)		Any service use	General practice	Counselling	Mental health service	Medication
Complete case analysis						
Model 1 (*n* = 2,649)	University attendance	0.54 (0.40–0.72)	0.49 (0.36–0.67)	0.58 (0.40–0.84)	0.66 (0.43–1.01)	0.36 (0.17–0.75)
Model 2 (*n* = 1,949)	+ Predisposing factors^a^	0.44 (0.30–0.63)	0.38 (0.26–0.57)	0.49 (0.31–0.79)	0.67 (0.39–1.12)	0.41 (0.26–0.66)
Model 3 (*n* = 1,935)	+ Family composition^b^	0.47 (0.32–0.68)	0.42 (0.28–0.62)	0.51 (0.32–0.83)	0.69 (0.41–1.16)	0.45 (0.28–0.71)
Model 4 (*n* = 991)	+ Enabling factors^c^	0.45 (0.14–0.93)	0.39 (0.22–0.72)	0.40 (0.20–0.83)	0.60 (0.27–1.36)	0.35 (0.17–0.73)
Model 5 (*n* = 454)	+ Need factors^d^	0.37 (0.14–0.93)	0.28 (0.11–0.74)	0.28 (0.11–0.74)	0.43 (0.11–1.69)	0.31 (0.12–0.76)
Matched (*n* = 522)		0.43 (0.19–0.96)	0.48 (0.21–1.09)	0.34 (0.12–0.97)	0.43 (0.13–1.43)	0.32 (0.12–0.90)
Imputed datasets						
Model 1 (*n* = 2,649)	University attendance	0.54 (0.40–0.72)	0.49 (0.36–0.67)	0.58 (0.40–0.84)	0.66 (0.43–1.01)	0.49 (0.34–0.72)
Model 2 (*n* = 2,649)	+ Predisposing factors	0.51 (0.38–0.68)	0.47 (0.34–0.64)	0.56 (0.39–0.81)	0.63 (0.41–0.97)	0.47 (0.32–0.69)
Model 3 (*n* = 2,649)	+ Family composition	0.52 (0.39–0.71)	0.48 (0.35–0.66)	0.58 (0.40–0.84)	0.65 (0.42-1.00)	0.49 (0.33–0.72)
Model 4 (*n* = 2,649)	+ Enabling factors	0.52 (0.39–0.71)	0.49 (0.35–0.68)	0.58 (0.39–0.87)	0.69 (0.43–1.08)	0.51 (0.34–0.76)
Model 5 (*n* = 2,649)	+ Need factors	0.58 (0.42–0.79)	0.54 (0.39–0.76)	0.65 (0.43–0.97)	0.78 (0.49–1.24)	0.57 (0.38–0.86)

Legend: a = sex assigned at birth, sexual orientation age 23, ethnicity at birth; b = family composition at birth; c = maternal education and neighbourhood deprivation based on IMD (Index of Multiple Deprivations) quintiles both at gestation; d = sMFQ (Short Mood and Feelings Questionnaire) scores at 17, SCDC (Social and Communications Difficulties Checklist) scores at 16, self-declared disability at 18

In Model 5, the only other variables with a notable association with health service use at age 24, after accounting for other confounders, were probable depression at age 17 (aOR = 3.28[95%CI:1.30;8.29], *p* = 0.01) and above-threshold autistic traits at age 16 (aOR = 4.44[95%CI:1.34;14.72], *p* = 0.02).

After matching, the odds of health service use before age 24 for a mental health problem was 57% lower among university attendees compared to non-attendees (OR = 0.43[95%CI:0.19;0.96], *p* = 0.04). Overall, there was a trend of lower odds of health service use across all secondary outcomes in university attendees compared to non-attendees, but no statistical evidence for the general practice and mental health service outcome.

In the imputed datasets, university attendees had about half the odds of reporting health service use by age 24 compared to non-attendees, even after adjusting for all confounders in Model 5 (aOR = 0.58[95%CI:0.42;0.79], *p* = 0.001). Consistent with the complete case analysis, there was a trend of lower odds of health service use across all secondary outcomes in university attendees compared to non-attendees, but no statistical evidence for the mental health service outcome.

When modelling the association only in individuals with probable depression, there was no evidence for an association between university attendance and health service use for a mental health problem (OR = 0.71[95%CI:0.38;1.31], *p* = 0.27) (see Online Resource 11).

### Effect modification

There was an indication of an effect modification of university attendance on health service use for a mental health problem by age 24 based on sexual orientation (*p* = 0.05) and maternal education (*p* < 0.001) in unadjusted models in complete case analysis (see Table 4 for full details). For individuals identifying with a minoritised sexual orientation, attending university was associated with lower odds of using a health service for a mental health problem (OR = 0.29[95%CI:0.17;0.49], *p* < 0.001) relative to their heterosexual peers (OR = 0.59[95%CI:0.38;0.92], *p* = 0.02). Among individuals whose maternal education level was non-compulsory university attendance was associated with lower odds of using a health service for a mental health problem (OR = 0.22[95%CI:0.09;0.51], *p* < 0.001), relative to those whose maternal education level was compulsory (OR = 0.63[95%CI:0.43;0.93], *p* = 0.02) (see Online Resources 12 for interaction plots).

**Table 4:** Analyses modelling the interactions between university attendance status and sociodemographic factors on health service use by age 24

	Any Health Service Use– Complete Case Analysis						Any Health Service Use– Imputed Datasets					
	Unadjusted models		Adjusted models			Unadjusted models			Adjusted models			
Correlates	N	OR (95% CI)		N	aOR (95% CI)		N	OR (95% CI)		N	aOR (95% CI)	
Interaction (university attendance X sex)	2,647	0.70 (0.35;1.39, *p* = 0.31)		1,380	0.75 (0.25;2.24, *p* = 0.60)		2,649	0.70 (0.35;1.40, *p* = 0.32)		2,649	0.72 (0.36;1.45, *p* = 0.36)	
Males												
Did not attend university		Reference			Reference			Reference			Reference	
Attended university		0.71 (0.39;1.30, *p* = 0.26)			0.83 (0.31;2.20, *p* = 0.71)			0.71 (0.39;1.29, *p* = 0.26)			0.78 (0.42;1.44, *p* = 0.43)	
Females												
Did not attend university		Reference			Reference			Reference			Reference	
Attended university		0.50 (0.36;0.69, *p* < 0.001)			0.62 (0.37;1.04, *p* = 0.07)			0.50 (0.36;0.69, *p* < 0.001)			0.56 (0.40;0.79, *p* < 0.001)	
Interaction (university attendance X ethnicity)	2,370	0.13 (0.01;1.30, *p* = 0.08)		NA	NA		2,649	0.24 (0.03;1.92, *p* = 0.18)		2,649	0.30 (0.04;2.41, *p* = 0.26)	
White												
Did not attend university		Reference			NA			Reference			Reference	
Attended university		0.59 (0.42;0.82, *p* = 0.002)			NA			0.56 (0.42;0.76, *p* < 0.001)			0.63 (0.47;0.86, *p* = 0.003	
Not White												
Did not attend university		Reference			NA			Reference			Reference	
Attended university		0.08 (0.01;0.75, *p* = 0.03)			NA			0.14 (0.02;1.04, *p* = 0.06)			0.19 (0.02;1.47, *p* = 0.11)	
Interaction (university attendance X sexual orientation)	2,167	0.50 (0.25;0.99, *p* = 0.05)		1,195	0.45 (0.17;1.23, *p* = 0.12)		2,649	0.62 (0.32;1.19, *p* = 0.15)		2,649	0.66 (0.34;1.29, *p* = 0.23)	
Heterosexual												
Did not attend university		Reference			Reference			Reference			Reference	
Attended university		0.59 (0.38;0.92, *p* = 0.02)			0.76 (0.39;1.48, *p* = 0.41)			0.61 (0.41;0.90, *p* = 0.01)			0.67 (0.45;0.99, *p* = 0.04)	
Minoritized sexual orientation												
Did not attend university		Reference			Reference			Reference			Reference	
Attended university		0.29 (0.17;0.49, *p* < 0.001)			0.34 (0.16;0.73, *p* = 0.005)			0.38 (0.23;0.62, *p* < 0.001)			0.44 (0.27;0.74, *p* = 0.002)	
Interaction (maternal education X university attendance)	2,199	0.35 (0.14;0.88; *p* < 0.001)		1,380	0.27 (0.07;1.05, *p* = 0.06)		2,649	0.34 (0.19;0.66, *p* < 0.001)		2,649	0.36 (0.19;0.68, *p* = 0.002)	
Compulsory education only												
Did not attend university		Reference			Reference			Reference			Reference	
Attended university		0.63 (0.43;0.93, *p* = 0.02)			0.88 (0.50;1.55, *p* = 0.66)			0.84 (0.56;1.28, *p* = 0.42)			0.94 (0.62;1.44, *p* = 0.78)	
Non-compulsory education												
Did not attend university		Reference			Reference			Reference			Reference	
Attended university		0.22 (0.09;0.51, *p* < 0.001)			0.24 (0.07;0.82, *p* = 0.02)			0.29 (0.19;0.46, *p* < 0.001)			0.33 (0.21;0.53, *p* < 0.001)	

Legend: adjusted models = adjustment for depression score reported at age 17, autistic traits reported at age 16

Note 1: NA indicates insufficient power estimate the interaction between university attendance and ethnicity after adjusting for confounders

Note 2: all interactions models conducted as separate analyses

In imputed dataset analyses, modification of effect was evident based on maternal highest education in unadjusted (*p* < 0.001) and adjusted models (*p* = 0.002). For individuals whose maternal education was non-compulsory, university attendance was associated with lower odds of using a health service (OR = 0.29[95%CI:0.19;0.46], *p* < 0.001; aOR = 0.33[0.21;0.53], *p* < 0.001) relative to individuals whose maternal education level was compulsory (OR = 0.84[95% CI: 0.56;1.28], *p* = 0.42; aOR = 0.94[95% CI: 0.62;1.44], *p* = 0.78).

## Discussion

In this study, we found evidence that individuals who attended university between the late 2000s and the late 2010s were less likely to use a health service for a mental health problem by age 24 compared to non-students. This association persisted across specific types of service utilization, except mental health services. The robustness of these findings was confirmed through various analytic methods. The overall percentage of university attendees using health services aligns closely with figures reported in another UK study [26]. Additionally, our analysis found evidence of a differential impact of university attendance on health service utilization for a mental health problem based on sexual orientation and maternal education levels.

### Findings in the context of existing evidence

Despite the worrying increase in young adults generally reporting symptoms of mental disorders, our findings suggest that university attendees are less likely to use health services for a mental health problem. This is evidenced by our observation that university attendees had lower odds of using a health service for a mental health problem by age 24 compared to non-attendees. We observed university attendees reporting depression symptoms at roughly half the rate of non-attendees at age seventeen. This finding broadly aligns with analyses of UK-based population cohort studies. For instance, these studies have demonstrated either equivalent or fewer mental health symptoms on average in student compared to non-student populations [13–15]. Differences between the prevalence of mental health symptoms in these studies and ours may be reflective of the varied instruments, cohorts, and time points utilised. Taken together, these findings suggest observed differences in health service use for a mental health problem may be due to differences in average prevalence of mental health symptoms between these two groups.

Another potential explanation of the findings of this study is that university attendance might offer some protection for mental health at the population level once at university. One factor could be related to cognitive skills and flexibility, potentially higher on average among university attendees. Population-based studies have found a negative correlation between these traits and psychopathology in general, as well as specific disorders [44, 45]. Although marginal, meta-analyses of quasi-experimental evidence suggest that each additional year of education marginally increases IQ [46], providing a substantial protective effect on the mental health of university attendees at the population level [45]. Any protective effect is likely to be multifaceted, potentially including the sense of purpose university attendance gives to young adults’ lives, opportunities to develop effective psychological coping strategies, encouragement of pro-social behaviours through civic engagement, social support, and role models who support development in the face of adversity [10, 47]. The occupational advantages which accrue from university attendance may also play a part [11, 48]. For individuals not attending university, exposure to these factors may be more variable, depending on the diverse environments in which they live and work.

An additional explanation for the key finding of this study could be that university students, on average, encounter more barriers to accessing services compared to non-students. Our findings diverge from the only international comparative study, which reported a higher prevalence of minimally adequate treatment among students than non-students [23]. This discrepancy may indicate that students face significant obstacles in early help-seeking and accessing treatment, yet once in treatment, they may be more likely to continue compared to non-attendees. Qualitative research has identified specific barriers to healthcare access for students, such as challenges associated with being registered at two addresses during university, which complicates access to secondary care, a tendency to value self-reliance, and stigma that may hinder primary care access [18–22]. Despite observing little evidence of a difference between attendees and non-attendees in the use of mental health services, this outcome was reported less frequently than other outcomes, and we observed a similar direction of effect. Given the low frequency of mental health service use reported in the data, future research could target recruitment of people with more severe mental health needs to examine difference in access between students and non-students.

Our analysis revealed evidence regarding the differential impact of university attendance on mental health service utilization based on individual characteristics. Specifically, university attendees with a minoritized sexual orientation and non-compulsory maternal education had lower odds of using health services compared to their non-attending counterparts. Echoing this, a recent international cross-sectional study, although limited to a student sample, identified similar patterns of past 12-month service utilization related to lower parental education, female gender, and heterosexual orientation [24]. Further investigations into the interaction between university attendance and individual characteristics affecting health service use should be conducted.

### Limitations

The findings of this study should be interpreted with consideration of several limitations. Firstly, although based on a population-based cohort study, the analytic sample was restricted to individuals with complete data on both the exposure and outcomes of interest. This restriction may have led to two potential limitations. First, the analytic sample might be ‘healthier’ than the general UK population, which could mean that both health service use and mental health symptoms are lower than what is seen in the broader population. This poses challenges for generalizability, and therefore, caution is advised when applying these findings to other populations. The second limitation related to the sample is the potential introduction of selection bias. This could occur if those who left the study before the exposure and outcomes were measured, differed systematically from those who remained. For instance, it’s possible that university attendees were more likely to stay in the study and might be healthier compared to the non-attendees who continued to participate. Despite adjusting for confounders and employing propensity score matching, selection bias introduced through lost-to-follow-up and sample selection cannot be controlled for.

Four limitations relate to the measurement of the exposure, outcome and confounder variables, which limit causal interpretation of the findings. First, the variable representing health service utilization for a mental health problem was based on self-report, and participants may have interpreted the recall period differently. Although derived from a validated interview schedule, this approach might result in an underestimation of actual service utilization. Second, individuals reported on health services at any point before age 24. Therefore, it is not possible to determine whether health service use took place before, during or after university attendance, introducing the possibility of reverse causality. Third, we were only able to include a measure of depressive symptoms for mental health scores. Therefore, differences in the observed association could be accounted for by differences in unmeasured symptoms or other reasons for health service presentations, including somatic concerns. Fourth, we assumed sexual orientation and carer status, recorded respectively at ages 22 and 23, were reflective of status prior to attending university. Carer status is likely to be time dependent, potentially violating this assumption.

Further limitations relate to the sample size, multiple imputation, student populations not included in the dataset, potential cohort effects and the measurement of the exposure. The study’s sample size may not have been sufficient to fully investigate effect modification; hence, the results from these analyses should be viewed as preliminary and warrant further exploration in other datasets. Regarding multiple imputations, we did not include interaction terms in the model. Therefore, these findings should be interpreted cautiously, as their lack of inclusion could bias the estimates in these models [49]. International students are not included in the ALSPAC cohort. Next, the individuals in this sample attended university during the 2010s, before significant shifts in mental disorder prevalence, help-seeking behaviour, and events such as the Covid-19 pandemic. Therefore, these analytical methods should be replicated in a more recent cohort study when such data becomes available. Finally, due to limitations in measuring university attendance, we were unable to distinguish between individuals who did not complete their degrees and those who completed shorter courses. Consequently, our sample may inadvertently include students who left university without graduating. Recent research indicates that individuals who attend university but do not graduate typically experience, worse long-term psychological and economic outcomes compared to both graduates and those who never attend university [50, 51]. Therefore, it is possible that graduates are even less likely to utilise health services for a mental health problem than non-attendees, suggesting that our study may underestimate the true magnitude of the observed association. Future research should therefore explicitly investigate differences in health service utilisation for a mental health problem among graduates, ‘non-completers’, and individuals who have never attended university.

## Conclusion

This study finds that university attendance is associated with lower health service use for a mental health problem in emerging adulthood. Our analysis also provides evidence suggesting that the reality is more complex at an individual level, with differences observed by certain demographic characteristics between attendees and non-attendees. Future research should explore potential explanations for these differences in health service use for a mental health problem between, and within, university-attending and non-attending populations. The implications of this study support the ongoing policy actions at the local level, which aim to identify and address social inequalities in access to appropriate, need-based mental health support. There is a need to enhance mental health services in various settings and environments where young adults work and live to prevent further exacerbation of health inequalities and access disparities.

## Electronic supplementary material

Below is the link to the electronic supplementary material.

Supplementary Material 1

## Data Availability

Data supporting the findings of the study can be accessed via an application to the ALSPAC cohort study team here: https://proposals.epi.bristol.ac.uk/. Analysis code is available from: https://github.com/tgosborn/ALSPAC_TG/tree/main.
