# Supplementary material for: Is university attendance associated with differences in health service use for a mental health problem in emerging adulthood? Evidence from the ALSPAC population-based cohort
Source: Soc Psychiatry Psychiatr Epidemiol. 2025 May 19;60(9):2175–88. doi: 10.1007/s00127-025-02922-3 (PMC12378762; doi:10.1007/s00127-025-02922-3)
Supplement: Supplementary file 1 — Supplementary Material 1 [file 127_2025_2922_MOESM1_ESM.docx]

Supplementary Online Resources

Online resources 1: Original protocol submitted to ALSPAC


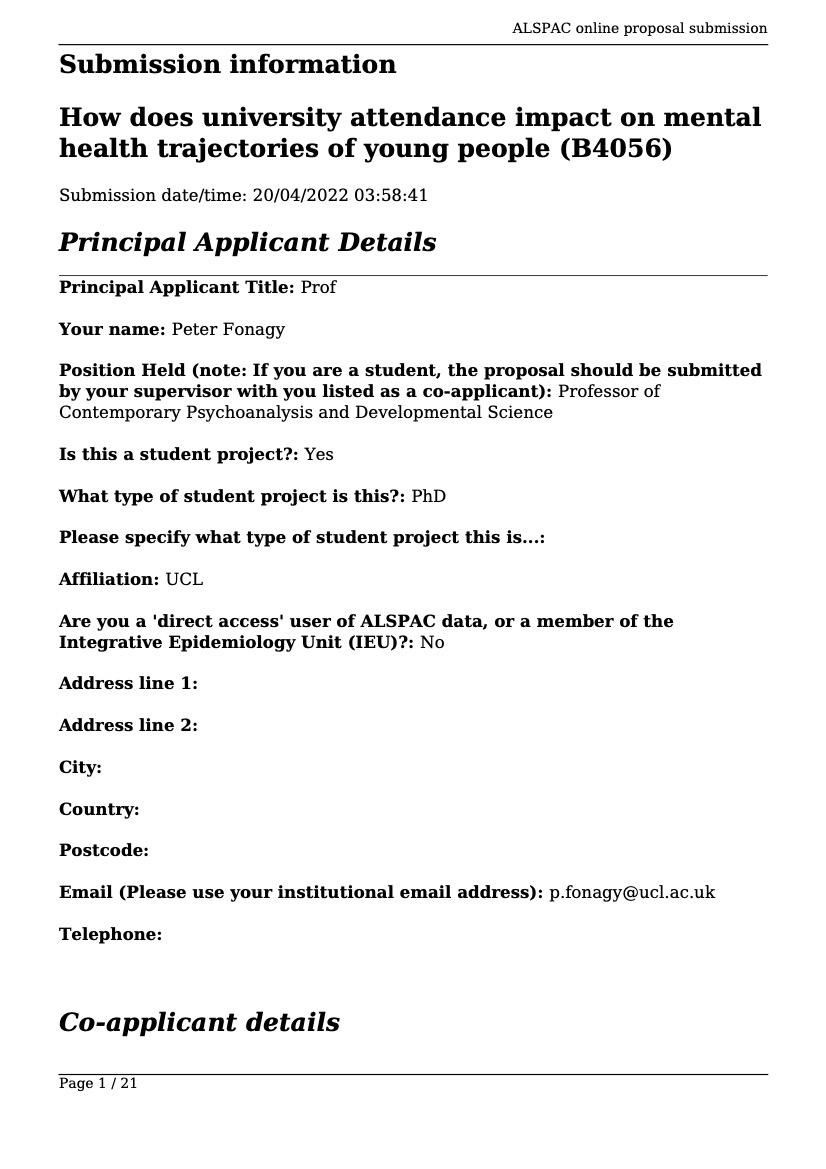


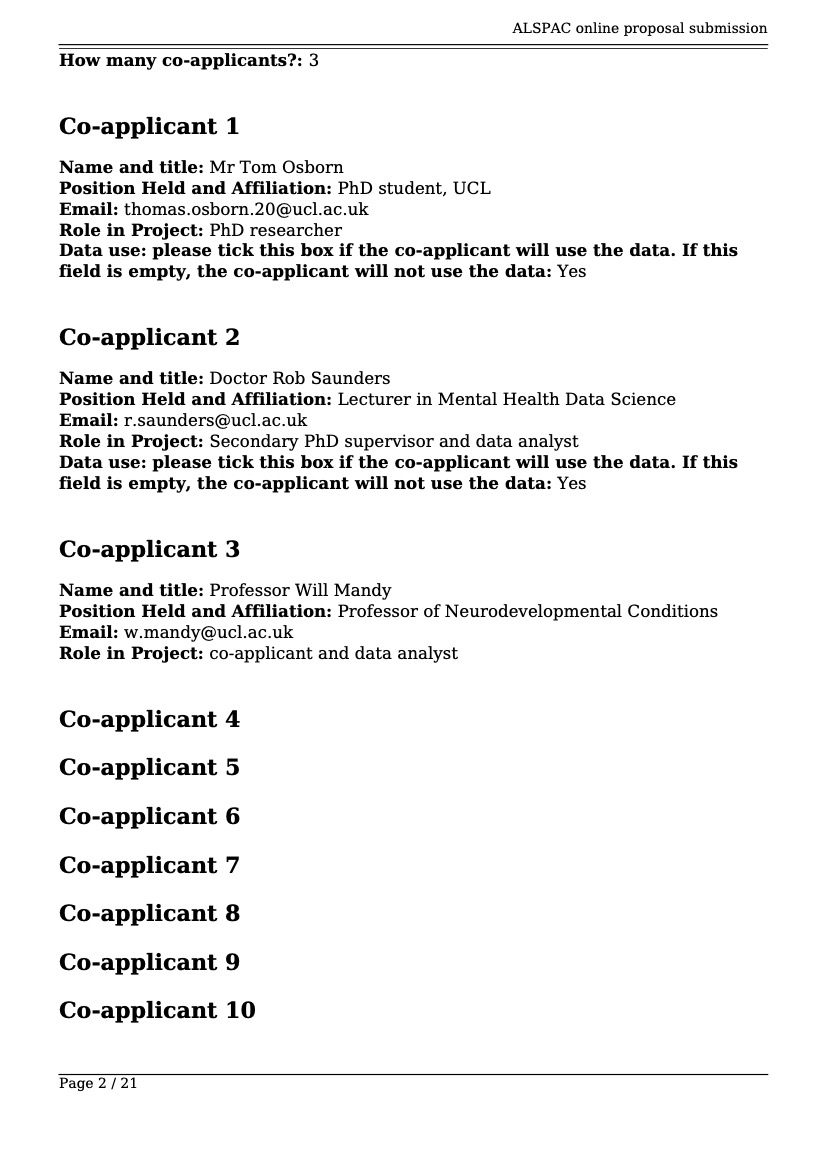


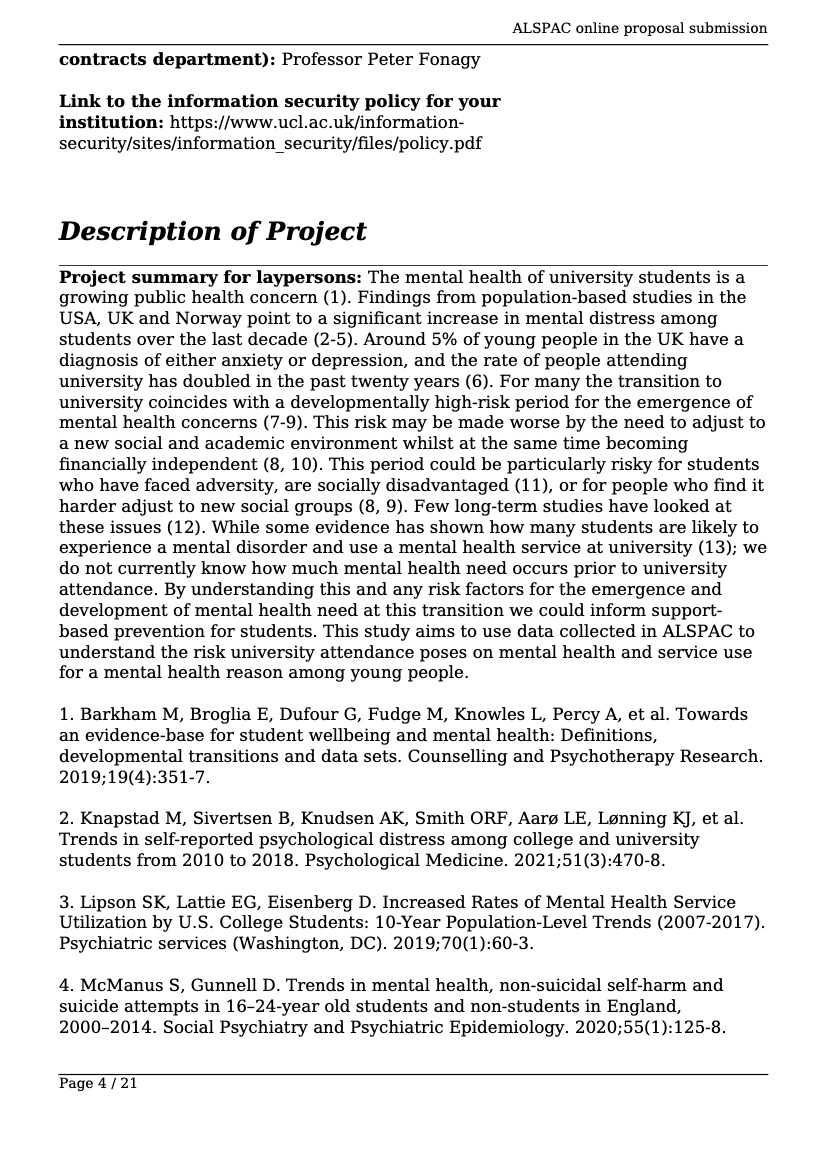


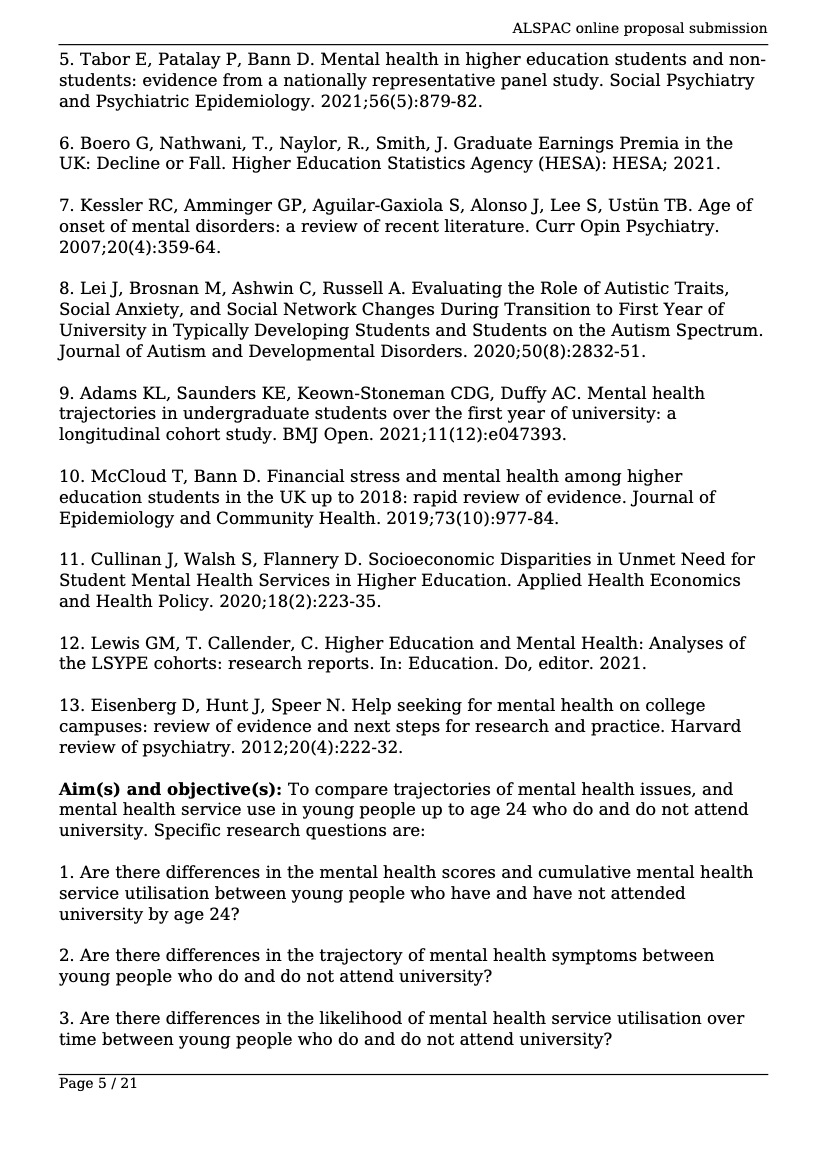


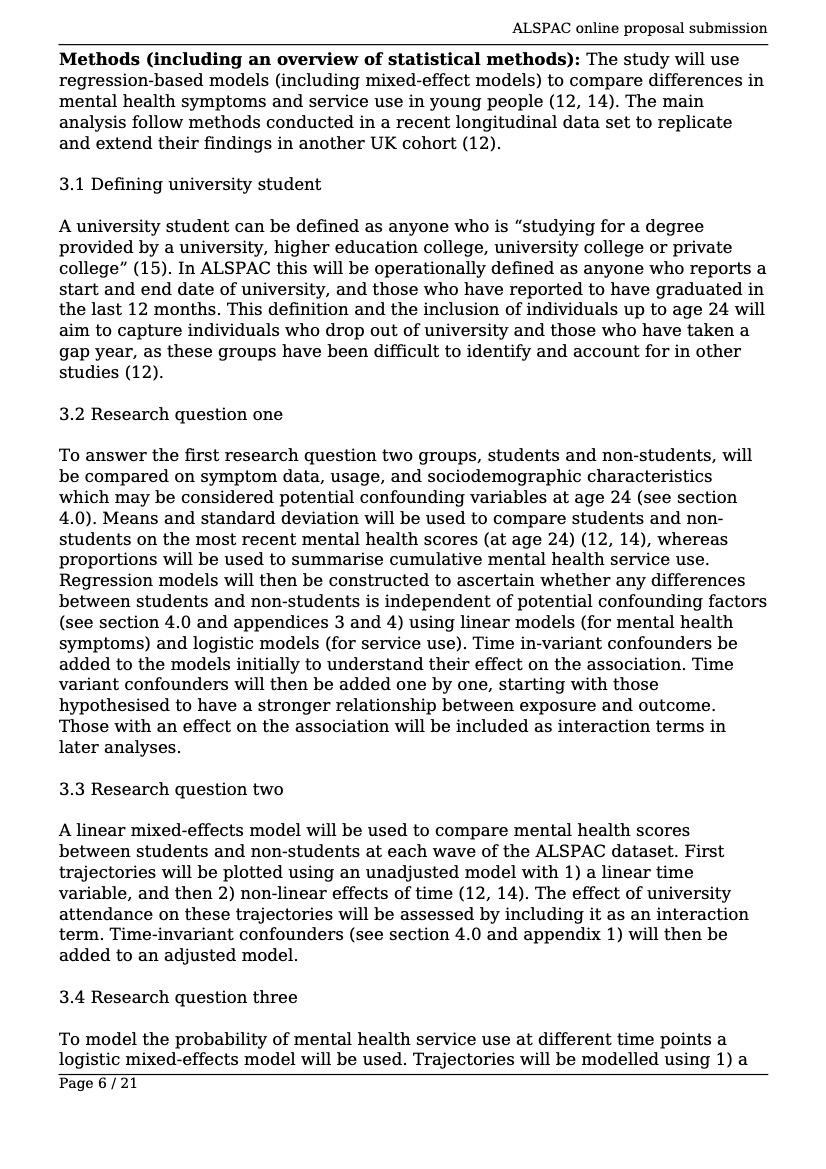


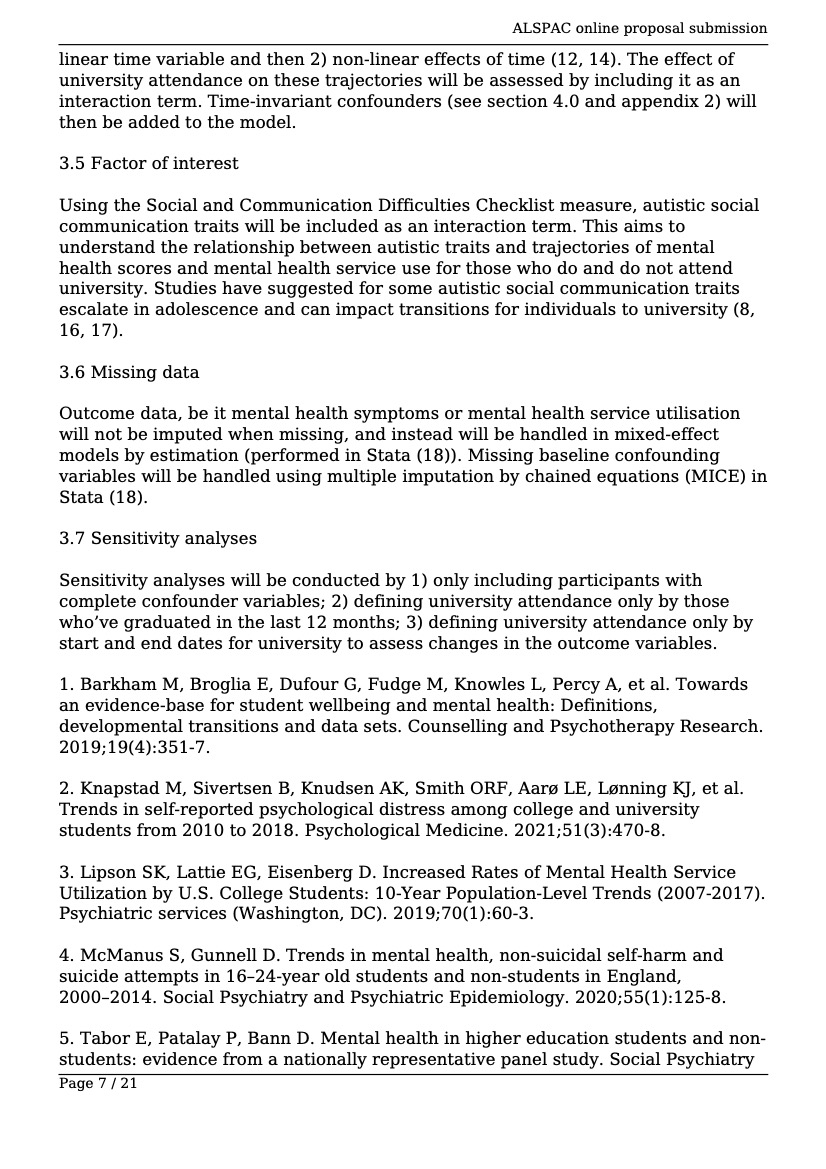


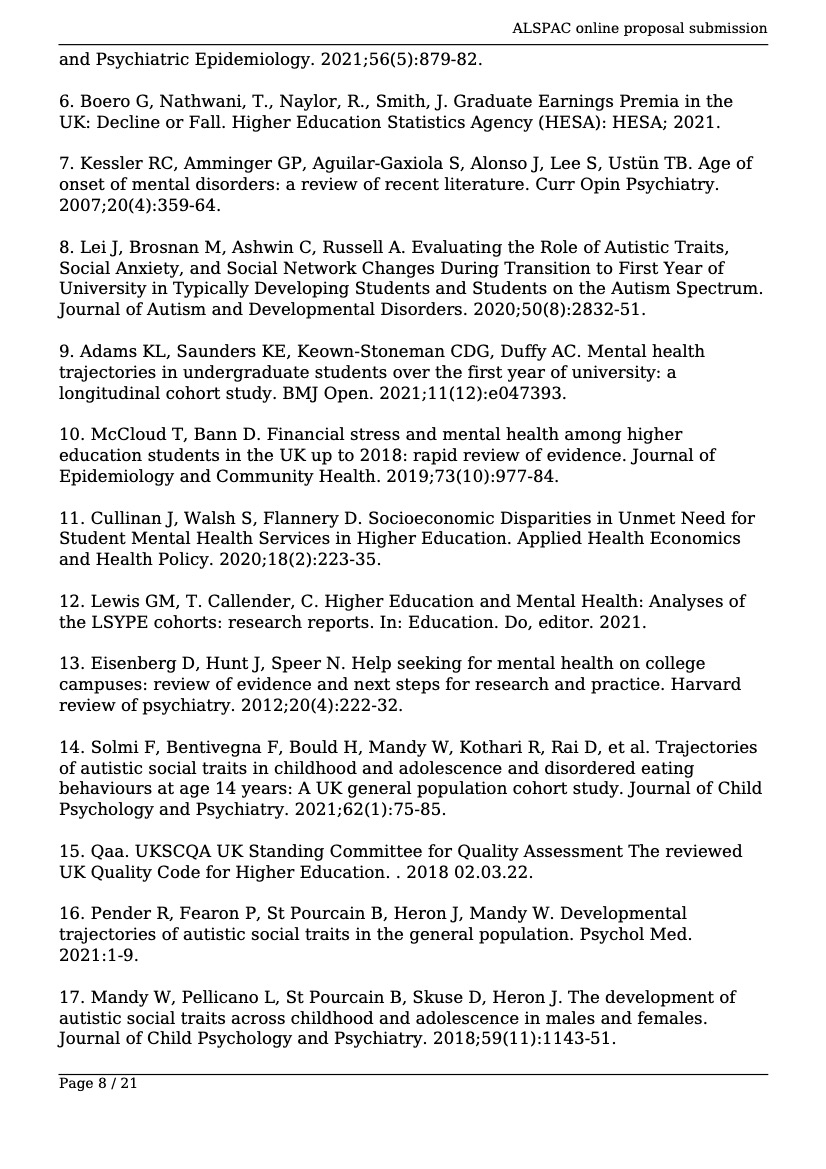


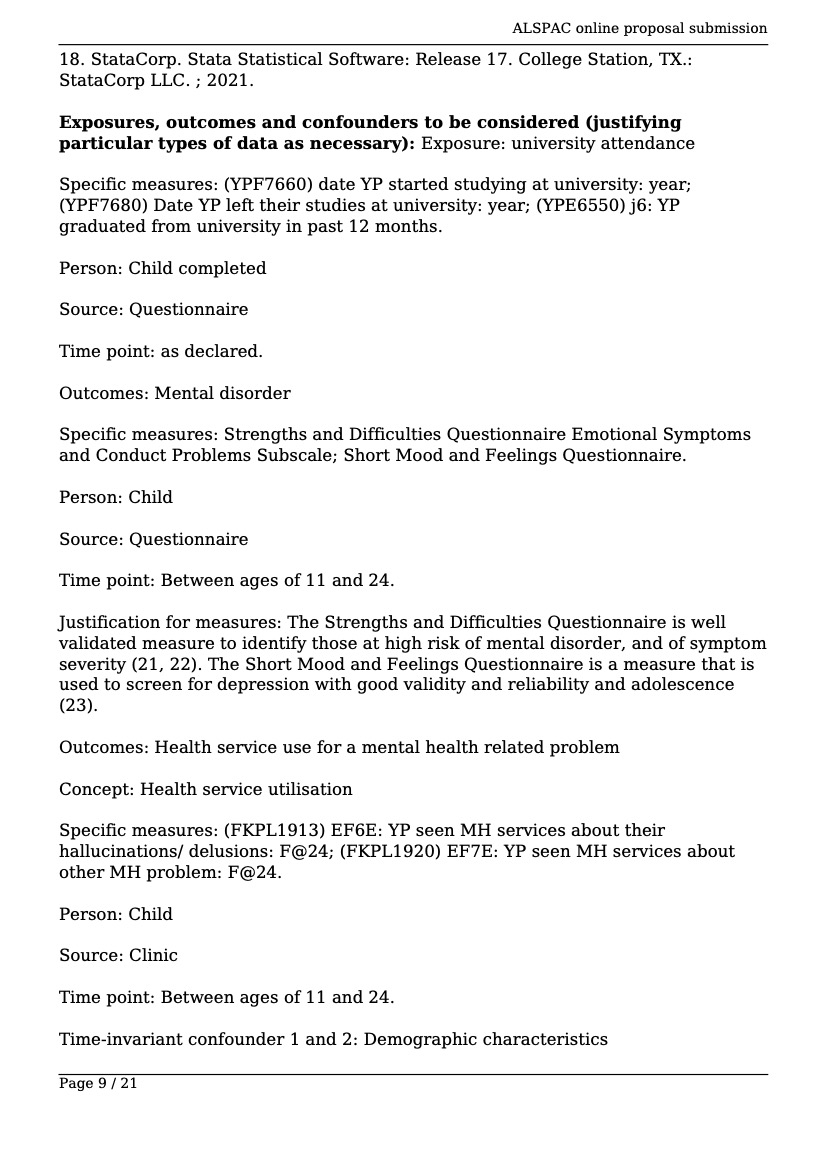


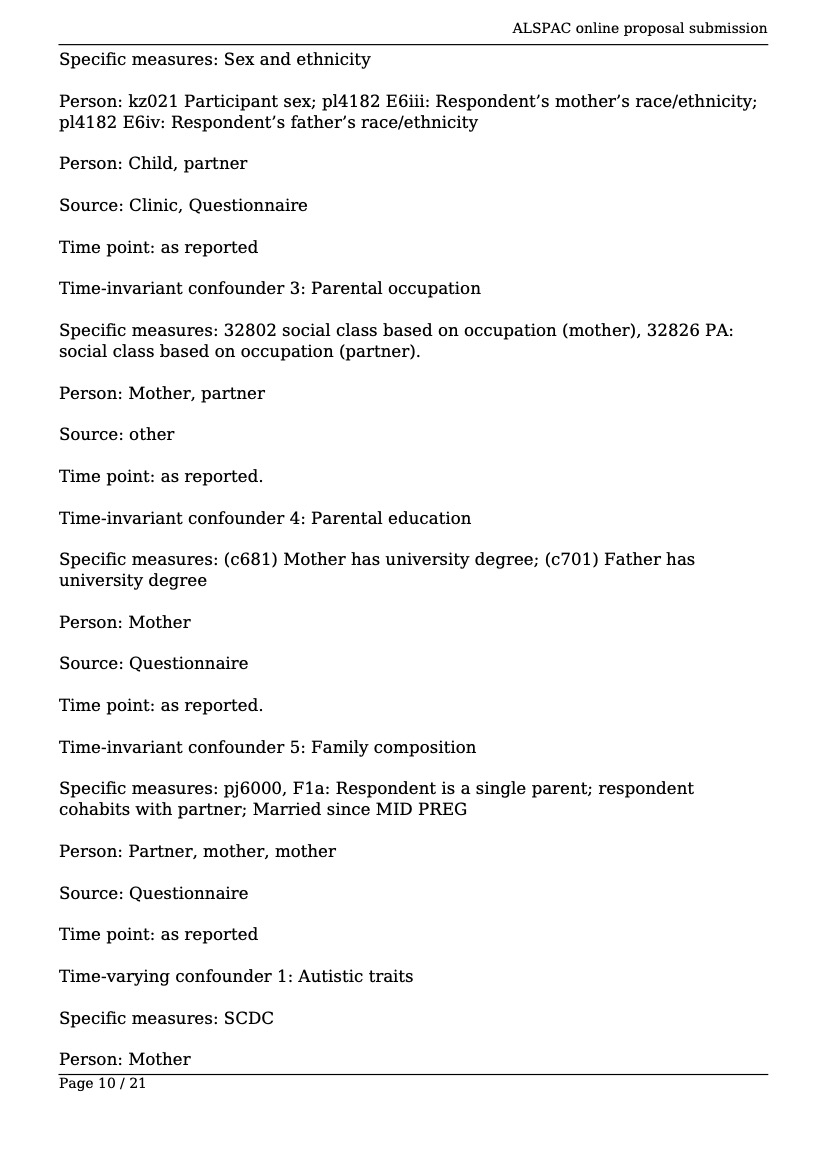


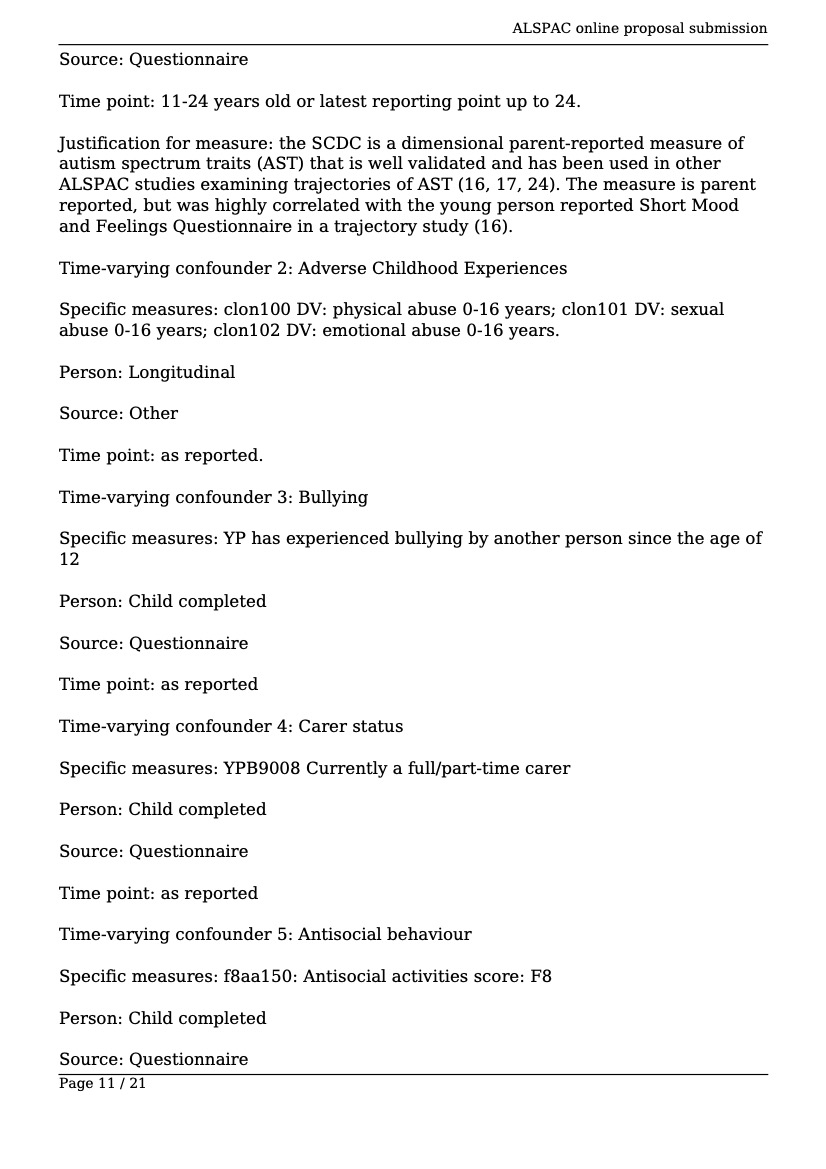


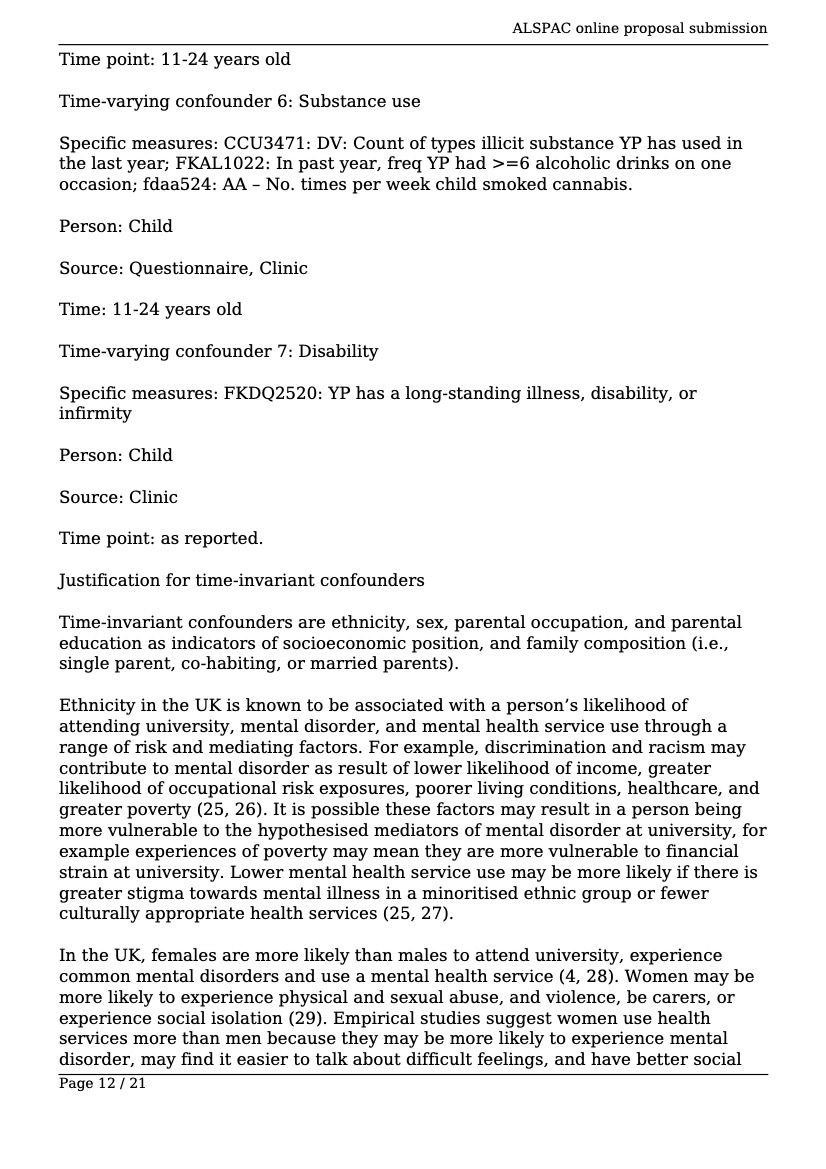


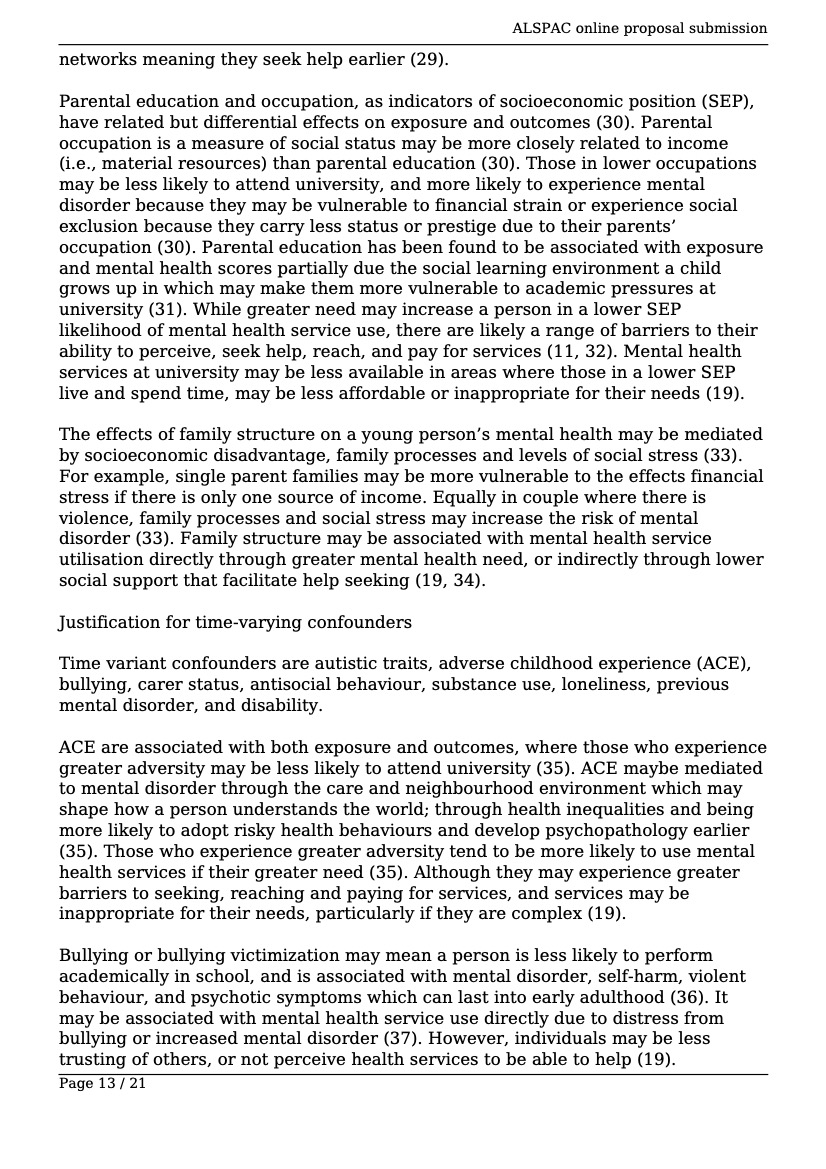


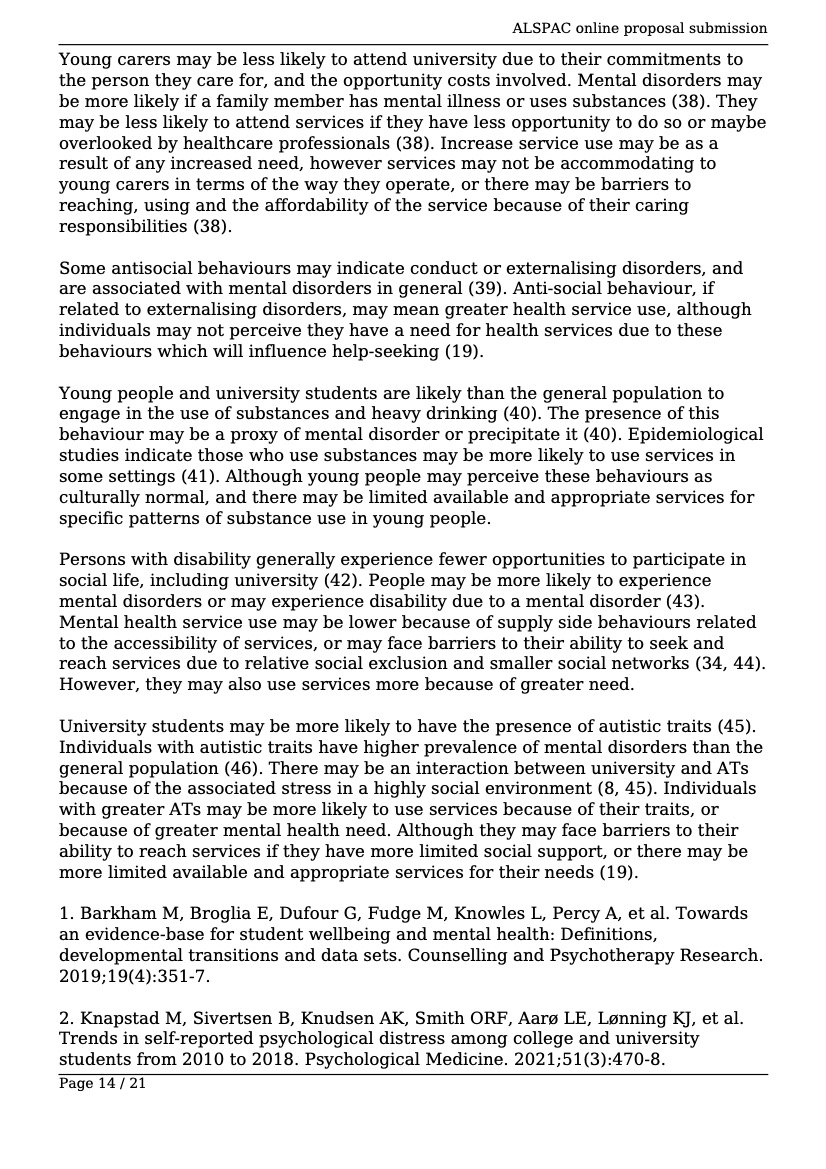


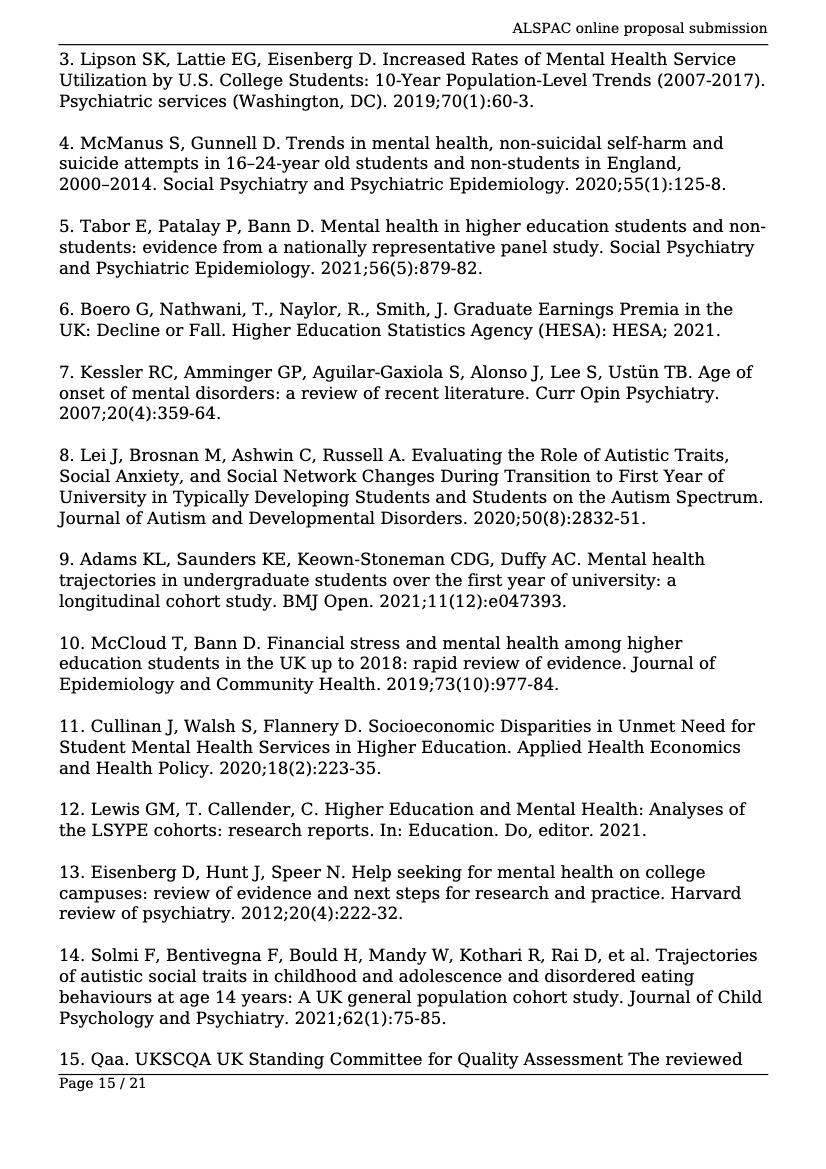


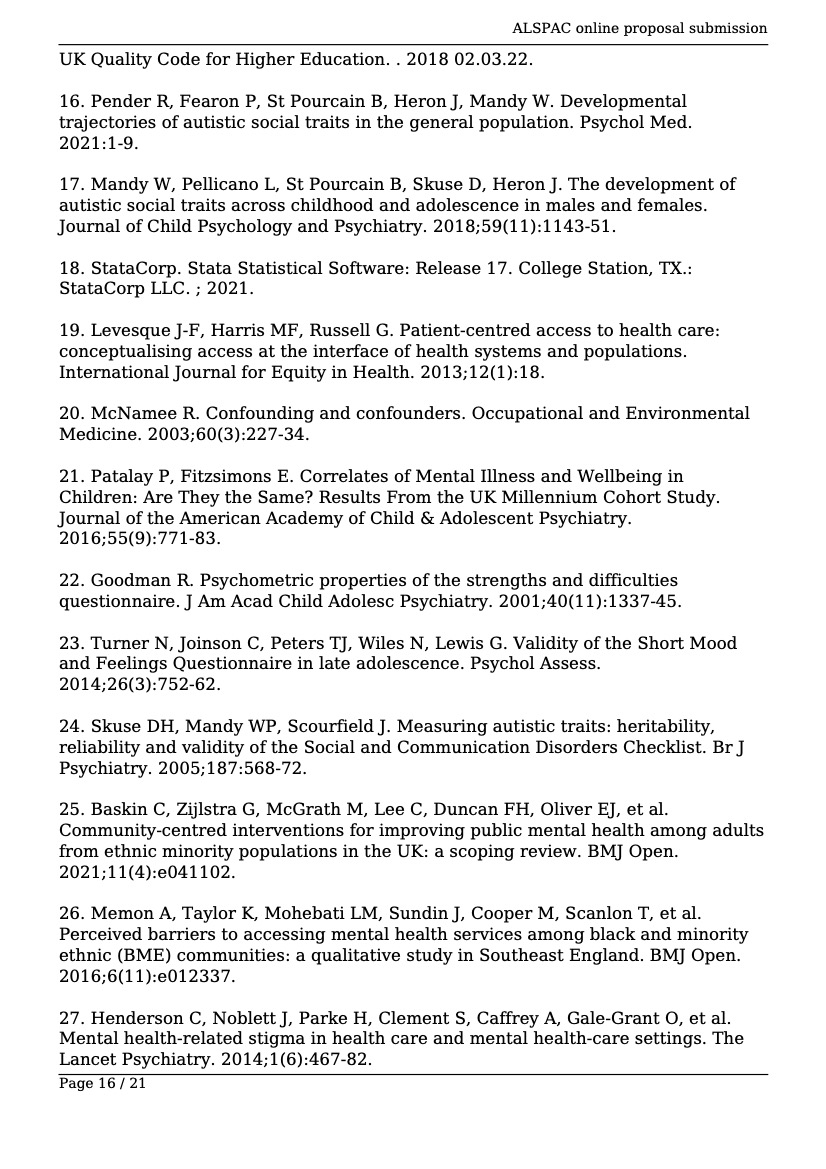


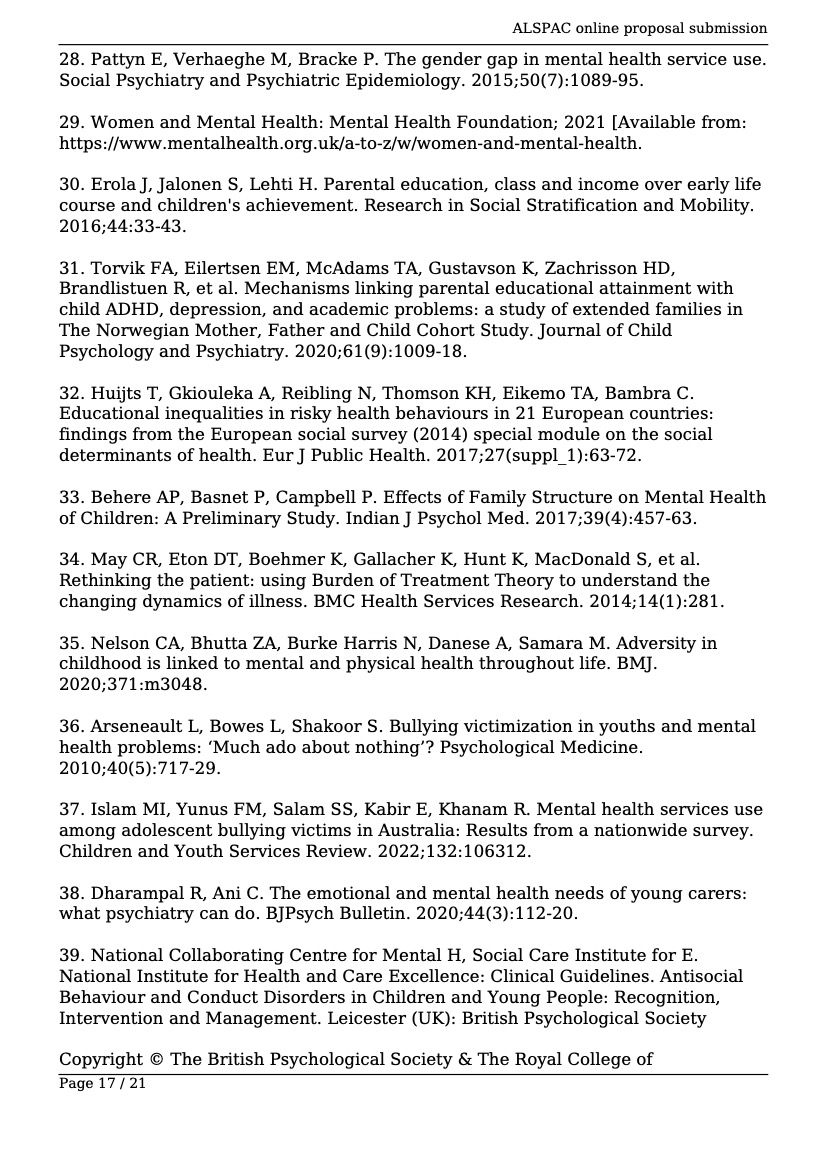


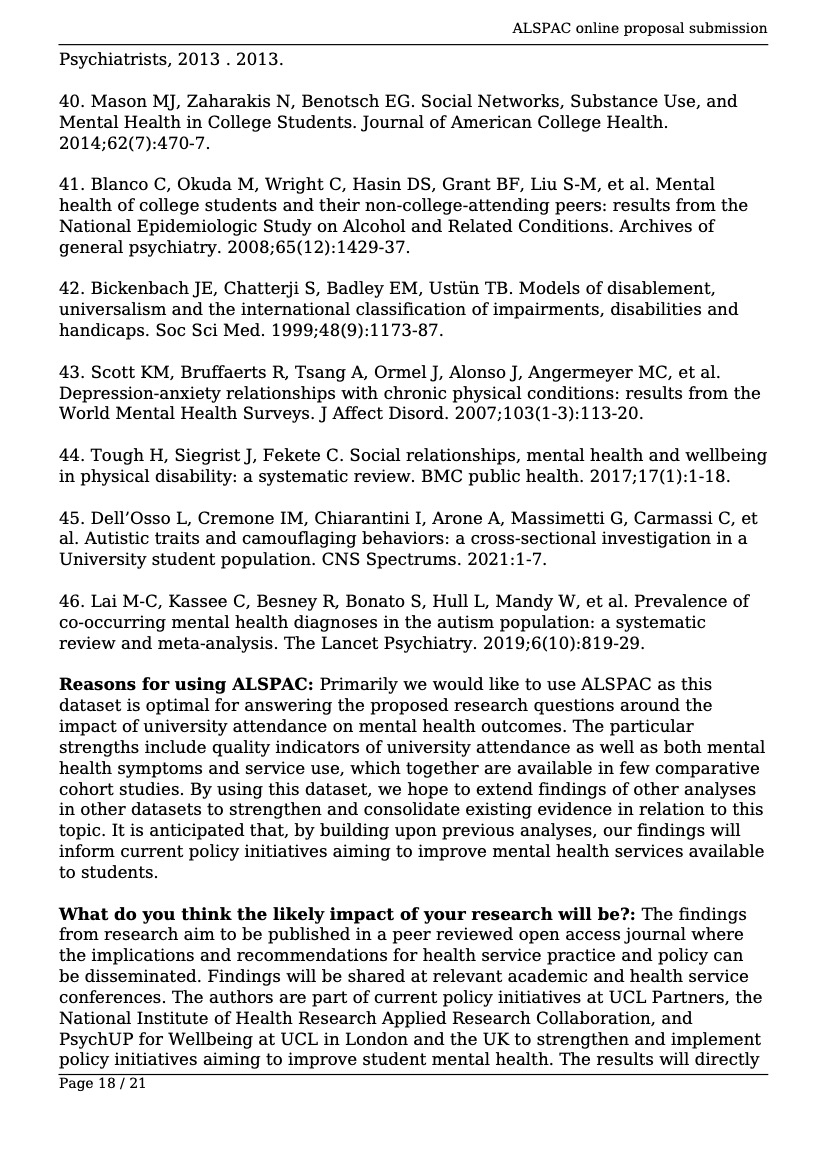


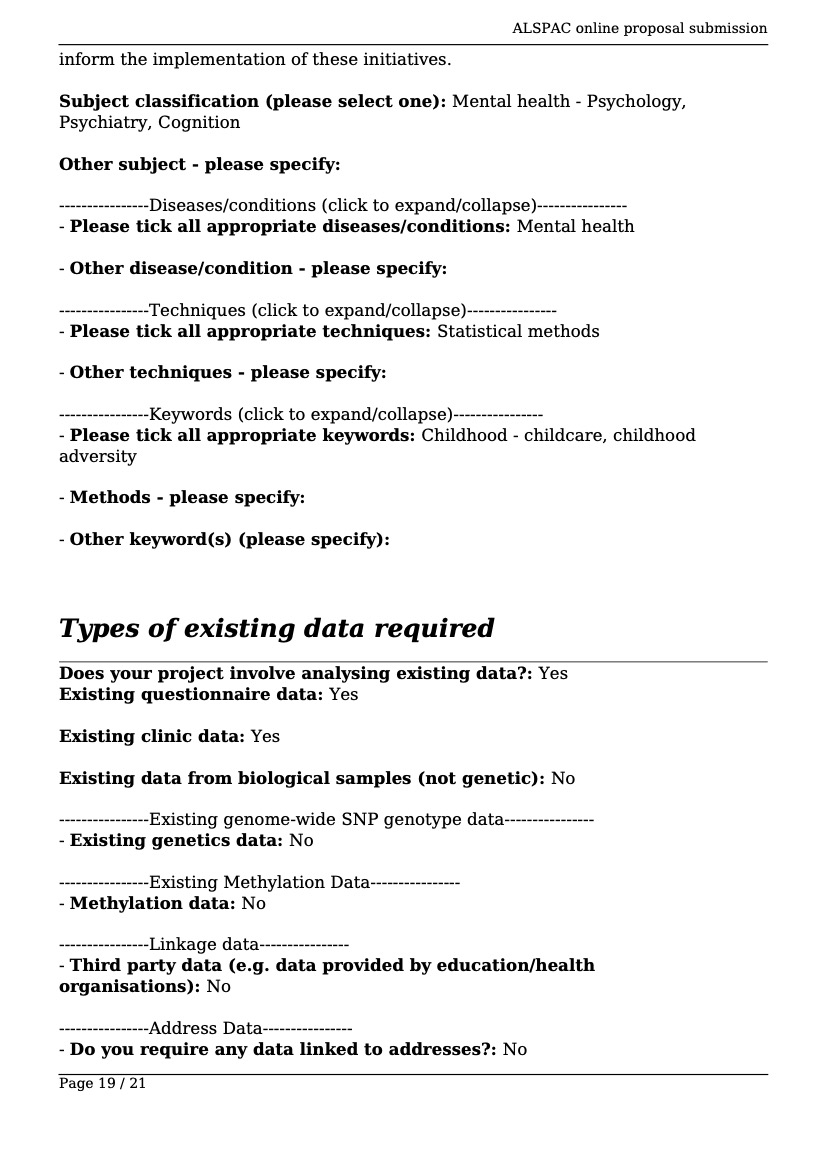


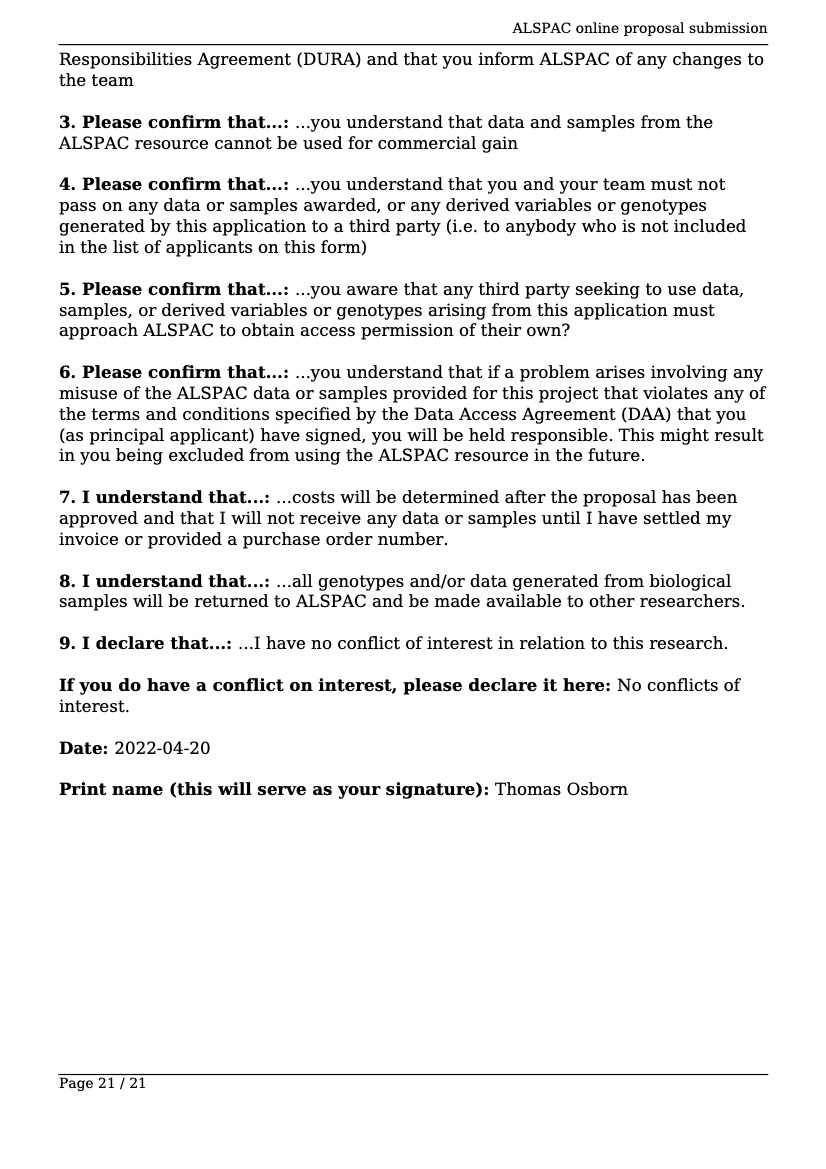

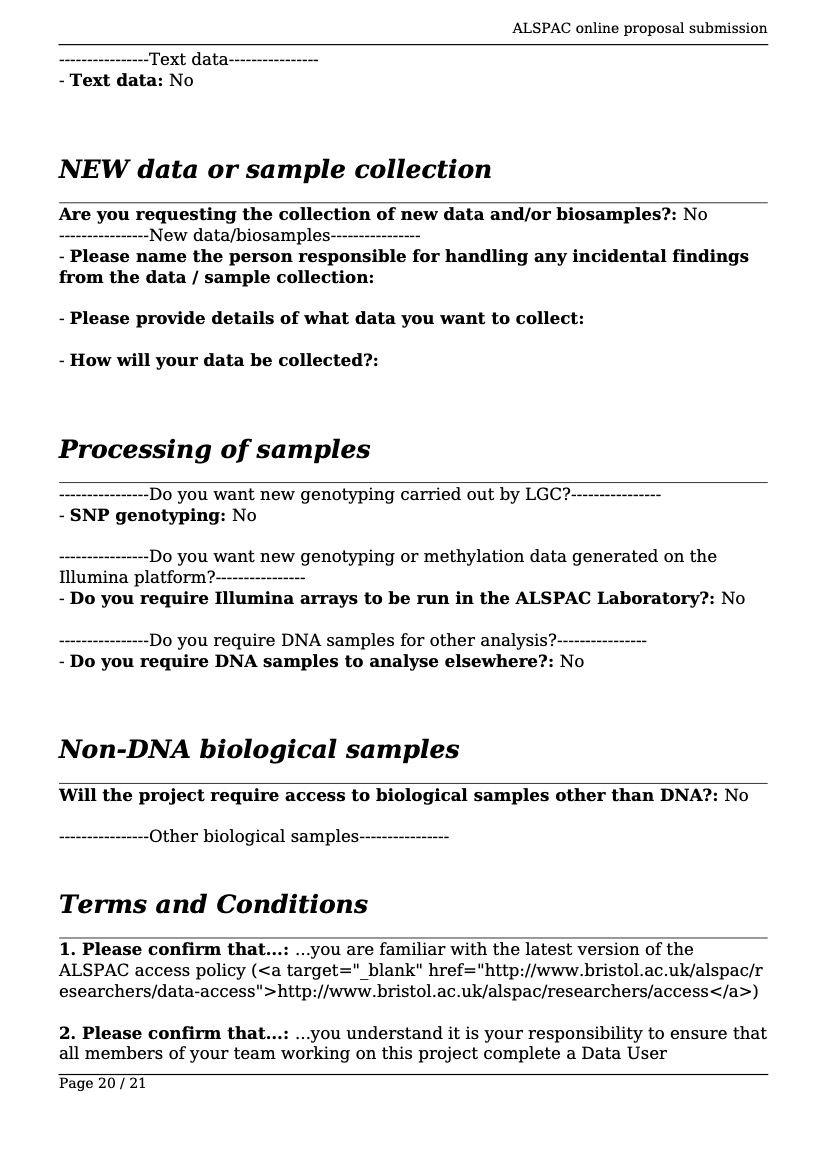


Deviations and changes from the proposal

The study deviates from the above proposal in the following ways. Firstly, the proposal included three separate analyses to be conducted. The study reports on analyses relating to the first research question and considers the outcome of mental health service use. This research question was attempted first for pragmatic reasons. Mental health scores (i.e., the short mood and feelings questionnaire) was included as a variable of interest in the analysis due to its robust association with the exposure and outcome of interest.

Substance use and antisocial behaviour was not included in the models because they were recorded antecedent to depression scores in the ALSPAC cohort. As depression scores were key variable of interest and the study aimed to see the effect of mental health problems on the exposure and outcome; substance use, and antisocial behaviour were not included due to their strong association and antecedency with depression scores which could have then led to the underestimation of the effect of depression scores on university attendance and mental health service use [1, 2] (Stringaris et al., 2014, Hammerton et al., 2023).

Parental education was included as indicator of individual social economic status. This has a robust association with the exposure and outcome and has been shown to be robust compared to class based on occupation and income [3, 4] (Reiss et al., 2019, Torvik et al., 2020). Parental education has been highly correlated with class and income [5] (Erola et al., 2016). Therefore, only parental education was only included as a confounder. Following the proposal submission, when requesting the data, the Index for Multiple Deprivation was included as this has an association with the exposure and outcome but provides an assessment of neighbourhood deprivation rather than individual social economic status.

Upon accessing the data after the proposal was submitted bullying it was apparent that bullying was part of the Adverse Childhood Experience variables collected in the cohort. Therefore, bullying is included in this variable. The Strengths and Difficulties Questionnaire was not reported by the child; therefore, the Short Mood and Feelings Questionnaire was used instead.

Sensitivity analyses were not conducted based on restricting the sample to individuals reporting graduation only or those based on end and start dates of university as the research question related to university attendance rather than graduation, drop out of or any other outcome. Missing data was imputed for all confounders as part of a sensitivity analysis.

Online resources 2: STROBE checklist

|  | **Item No.** | **Recommendation** | **Page  No.** | **Relevant text from manuscript** |
| --- | --- | --- | --- | --- |
| **Title and abstract** | 1 | (*a*) Indicate the study’s design with a commonly used term in the title or the abstract | 1 | Is university attendance associated with differences in health service use for a mental health problem in emerging adulthood? Evidence from the ALSPAC population-based cohort |
|  |  | (*b*) Provide in the abstract an informative and balanced summary of what was done and what was found | 2 |  |
| **Introduction** | | | |  |
| Background/rationale | 2 | Explain the scientific background and rationale for the investigation being reported | 3-4 |  |
| Objectives | 3 | State specific objectives, including any prespecified hypotheses | 4 |  |
| **Methods** | | | |  |
| Study design | 4 | Present key elements of study design early in the paper | 4 |  |
| Setting | 5 | Describe the setting, locations, and relevant dates, including periods of recruitment, exposure, follow-up, and data collection | 4 |  |
| Participants | 6 | (*a*) *Cohort study*—Give the eligibility criteria, and the sources and methods of selection of participants. Describe methods of follow-up  *Case-control study*—Give the eligibility criteria, and the sources and methods of case ascertainment and control selection. Give the rationale for the choice of cases and controls  *Cross-sectional study*—Give the eligibility criteria, and the sources and methods of selection of participants | 4-5 |  |
|  |  | (*b*) *Cohort study*—For matched studies, give matching criteria and number of exposed and unexposed  *Case-control study*—For matched studies, give matching criteria and the number of controls per case | 6-7 |  |
| Variables | 7 | Clearly define all outcomes, exposures, predictors, potential confounders, and effect modifiers. Give diagnostic criteria, if applicable | 5-6, Table 1 |  |
| Data sources/ measurement | 8* | For each variable of interest, give sources of data and details of methods of assessment (measurement). Describe comparability of assessment methods if there is more than one group | 5-6, Table 1 |  |
| Bias | 9 | Describe any efforts to address potential sources of bias | 6-7; 10-12 |  |
| Study size | 10 | Explain how the study size was arrived at | 4-7 |  |

| Quantitative variables | 11 | Explain how quantitative variables were handled in the analyses. If applicable, describe which groupings were chosen and why | 4-7, table 1 |  |
| --- | --- | --- | --- | --- |
| Statistical methods | 12 | (*a*) Describe all statistical methods, including those used to control for confounding | 6-7 |  |
|  |  | (*b*) Describe any methods used to examine subgroups and interactions | 6-7 |  |
|  |  | (*c*) Explain how missing data were addressed | 6-7 |  |
|  |  | (*d*) *Cohort study*—If applicable, explain how loss to follow-up was addressed  *Case-control study*—If applicable, explain how matching of cases and controls was addressed  *Cross-sectional study*—If applicable, describe analytical methods taking account of sampling strategy | 6-7 |  |
|  |  | (*e*) Describe any sensitivity analyses | 6-7, online resources |  |
| **Results** | | | | |
| Participants | 13* | (a) Report numbers of individuals at each stage of study—e.g., numbers potentially eligible, examined for eligibility, confirmed eligible, included in the study, completing follow-up, and analysed | 7-8 |  |
|  |  | (b) Give reasons for non-participation at each stage | 6-8, figure 1 |  |
|  |  | (c) Consider use of a flow diagram | 7, Figure 1 |  |
| Descriptive data | 14* | (a) Give characteristics of study participants (e.g., demographic, clinical, social) and information on exposures and potential confounders | 7-9 |  |
|  |  | (b) Indicate number of participants with missing data for each variable of interest | 7-8 |  |
|  |  | (c) *Cohort study*—Summarise follow-up time (e.g., average and total amount) | N/A |  |
| Outcome data | 15* | *Cohort study*—Report numbers of outcome events or summary measures over time | N/A |  |
|  |  | *Case-control study—*Report numbers in each exposure category, or summary measures of exposure | 7-9 |  |
|  |  | *Cross-sectional study—*Report numbers of outcome events or summary measures | N/A |  |
| Main results | 16 | (*a*) Give unadjusted estimates and, if applicable, confounder-adjusted estimates and their precision (e.g., 95% confidence interval). Make clear which confounders were adjusted for and why they were included | 8-9 |  |
|  |  | (*b*) Report category boundaries when continuous variables were categorized | 5-6 table 1 |  |
|  |  | (*c*) If relevant, consider translating estimates of relative risk into absolute risk for a meaningful time period | N/A |  |

Continued on next page

| Other analyses | 17 | Report other analyses done—e.g. analyses of subgroups and interactions, and sensitivity analyses | 7-9 |  |
| --- | --- | --- | --- | --- |
| **Discussion** | | | | |
| Key results | 18 | Summarise key results with reference to study objectives | 10 |  |
| Limitations | 19 | Discuss limitations of the study, taking into account sources of potential bias or imprecision. Discuss both direction and magnitude of any potential bias | 11-12 |  |
| Interpretation | 20 | Give a cautious overall interpretation of results considering objectives, limitations, multiplicity of analyses, results from similar studies, and other relevant evidence | 9-11 |  |
| Generalisability | 21 | Discuss the generalisability (external validity) of the study results | 11-12 |  |
| **Other information** | |  | | |
| Funding | 22 | Give the source of funding and the role of the funders for the present study and, if applicable, for the original study on which the present article is based | 13 |  |

Online resource 3: Comparison between the analytic sample and the excluded sample

| **Correlates** | **Analytic sample**  **N** | **%** | **Excluded sample**  **N** | **%** | χ^2^ | p-value |
| --- | --- | --- | --- | --- | --- | --- |
| Sex assigned at birth |  |  |  |  |  |  |
| Being male at birth | 859 | 32.45 | 6,829 | 55.13 |  |  |
| Being female at birth | 1,788 | 67.56 | 5,559 | 44.87 | 448.75 | <0.001 |
| Ethnicity |  |  |  |  |  |  |
| White | 2,286 | 96.46 | 9,234 | 94.58 |  |  |
| Minoritized | 84 | 3.54 | 529 | 5.42 | 13.96 | <0.001 |
| Sexual orientation |  |  |  |  |  |  |
| Heterosexual | 1,676 | 77.34 | 1,596 | 80.12 |  |  |
| Minoritized | 491 | 22.66 | 396 | 19.88 | 4.78 | 0.03 |
| Maternal highest education |  |  |  |  |  |  |
| Compulsory only | 1,670 | 75.94 | 6,672 | 86.08 |  |  |
| Above compulsory | 529 | 24.06 | 1,079 | 13.92 | 129.88 | <0.001 |
| Family composition at gestation |  |  |  |  |  |  |
| Married or cohabiting | 2,056 | 85.06 | 8,302 | 73.99 |  |  |
| Not married or co-habiting | 361 | 14.94 | 2,919 | 26.01 | 133.60 | <0.001 |
| IMD quintile at gestation |  |  |  |  |  |  |
| Least deprived | 461 | 33.63 | 1,615 | 23.99 |  |  |
| 2^nd^ least deprived | 247 | 18.02 | 1,079 | 16.03 |  |  |
| 3^rd^ least deprived | 234 | 17.07 | 1,099 | 16.33 |  |  |
| 4^th^ least deprived | 214 | 15.61 | 1,221 | 18.14 |  |  |
| Most deprived | 215 | 15.68 | 1,717 | 25.51 | 94.61 | <0.001 |
| Autistic traits at age 16 |  |  |  |  |  |  |
| Below threshold | 1,776 | 93.03 | 3,199 | 90.14 |  |  |
| Above threshold | 133 | 6.97 | 350 | 9.86 | 12.90 | <0.001 |
| sMFQ score at age 17 |  |  |  |  |  |  |
| < 12 | 1,635 | 84.23 | 2,040 | 79.94 |  |  |
| ≥ 12 | 306 | 15.77 | 512 | 20.06 | 13.67 | <0.001 |
| Disability at age 18 |  |  |  |  |  |  |
| A reported disability | 251 | 15.39 | 278 | 16.32 |  |  |
| No reported disability | 1,380 | 84.61 | 1,425 | 83.68 | 0.55 | 0.46 |
| Carer status at age 22 |  |  |  |  |  |  |
| Carer | 47 | 2.82 | 65 | 4.28 |  |  |
| Not a carer | 1,617 | 97.18 | 1,455 | 95.72 | 4.93 | 0.03 |
| Adverse childhood experiences between age 0-16 |  |  |  |  |  |  |
| 0 | 1,290 | 48.70 | 10,028 | 77.16 |  |  |
| 1 | 816 | 30.80 | 1,925 | 14.81 |  |  |
| ≥ 2 | 543 | 20.50 | 1,043 | 8.03 | 905.34 | <0.001 |

Online resource 4: Proportion of individuals reporting health service use and depression scores by included variables

|  | Attendees |  |  | Non-attendees |  |  |
| --- | --- | --- | --- | --- | --- | --- |
| Variable | Service use %  (95%CI) | sMFQ ≥ 12 %  (95%CI) | sMFQ  *M* (sd) | Service use %  (95%CI) | sMFQ ≥ 12 %  (95%CI) | sMFQ  *M* (sd) |
| Overall Sample | 6.5 (5.4-7.7) | 13.4 (11.9-15.1) | 6.0 (4.8) | 11.4 (9.3-13.9) | 25.8 (22.7-29.2) | 7.6 (6.1) |
| Sex assigned at birth |  |  |  |  |  |  |
| Female | 7 (5.8-8.5) | 15.0 (13.0-17.1) | 6.4 (4.9) | 13.3 (10.6-16.5) | 29.0 (25.2-33.2) | 8.3 (6.3) |
| Male | 5.3 (3.8-7.3) | 10.6 (8.4-13.3) | 5.4 (4.5) | 7 (4.6-11.4) | 18.6 (14.0-24.4) | 6.0 (5.2) |
| Ethnicity |  |  |  |  |  |  |
| White | 6.3 (5.3-7.6) | 13.2 (11.6-15.0) | 6.0 (4.8) | 10.2 (8.1-12.9) | 24.9 (21.6-28.5) | 7.4 (6.0) |
| Not white | 1.6 (0.2-10.7) | 16.1 (8.6-28.1) | 6.8 (5.2) | 17.4 (6.7-38.2) | 42.8 (26.2-61.3) | 9.3 (6.7) |
| Sexual Orientation |  |  |  |  |  |  |
| Heterosexual | 4.4 (3.4-5.7) | 10.4 (8.8-12.3) | 5.5 (4.4) | 7.3 (5.3-10.1) | 19.7 (16.2-23.8) | 6.7 (5.7) |
| Not heterosexual | 10.3 (7.6-13.8) | 22.2 (18.1-27.0) | 7.4 (5.4) | 28.3 (20.9-37.3) | 43.8 (34.6-53.4) | 10.5 (6.6) |
| Family composition at gestation |  |  |  |  |  |  |
| Married or co-habiting | 5.5 (4.5-6.8) | 12.7 (11.1-14.5) | 5.9 (4.6) | 9.9 (7.6-12.7) | 24.3 (20.8-28.2) | 7.3 (6.0) |
| Not married or co-habiting | 9.7 (6.4-14.3) | 17.8 (13.2-23.5) | 6.8 (5.6) | 13.4 (8.6-20.3) | 30.2 (23.2-38.3) | 8.2 (6.3) |
| Maternal highest education |  |  |  |  |  |  |
| Compulsory only | 6.2 (5-7.8) | 14.4 (12.4-16.6) | 6.2 (4.8) | 9.5 (7.2-12.3) | 13.9 (5.9-29.3) | 7.4 (5.9) |
| Non-compulsory | 5.7 (3.9-8.2) | 10.7 (8.2-13.7) | 5.5 (4.5) | 21.4 (11.5-36.3) | 25.1 (21.6-29.1) | 6.5 (4.8) |
| IMD^a^ quintile at gestation |  |  |  |  |  |  |
| Least deprived | 5.7 (3.8-8.6) | 10.4 (7.6-14.0) | 5.7 (4.5) | 9.6 (5.1-17.4) | 24.7 (16.7-40.0) | 7.1 (5.7) |
| 2^nd^ | 3.2 (1.5-7.0) | 11.6 (7.6-17.3) | 5.5 (4.8) | 9.2 (4.4-19.9) | 27.1 (18.0-38.7) | 7.4 (5.5) |
| 3^rd^ | 5.9 (3.2-10.7) | 16.2 (11.1-23.1) | 6.2 (5.2) | 9.4 (4.2-19.1) | 26.6 (17.2-38.7) | 7.6 (6.4) |
| 4^th^ | 5.1 (2.4-10.3) | 17.0 (11.8-24.0) | 6.2 (4.8) | 11.8 (6.3-21.2) | 17.2 (10.7-26.7) | 6.6 (6.1) |
| Most deprived | 8.2 (4.5-14.6) | 17.2 (11.3-25.2) | 7.0 (5.3) | 11.8 (6.7-20.1) | 28.7 (20.2-39.0) | 8.3 (6.3) |
| Disability at age 18 |  |  |  |  |  |  |
| Yes | 11.9 (8.0-17.4) | 17.9 (13.1-24.0) | 6.9 (5.3) | 21.2 (13.0-32.7) | 32.3 (22.1-44.5) | 8.8 (6.3) |
| No | 4.4 (3.3-5.8) | 10.9 (9.2-12.9) | 5.6 (4.5) | 9.4 (6.6-13.0) | 22.4 (18.3-27.1) | 7.1 (6.0) |
| sMFQ^b^ score at age 17 |  |  |  |  |  |  |
| < 12 | 4.6 (3.6-6.0) | N/A | N/A | 6 (4.0-9.0) | N/A | N/A |
| ≥ 12 | 14.2 (9.9-19.9) | N/A | N/A | 19 (12.8-27.1) | N/A | N/A |
| Autistic traits at age 16 |  |  |  |  |  |  |
| Below threshold | 5.0 (3.9-6.3) | 12.6 (10.9-14.4) | 5.8 (4.7) | 9.4 (7.0-12.6) | 24.3 (20.6-28.5) | 7.2 (5.6) |
| Above threshold | 17.3 (10.5-27.1) | 18.1 (11.1-27.8) | 7.5 (4.5) | 21.2 (12.1-34.3) | 35.3 (23.5-49-2) | 8.4 (7.0) |
| Adverse childhood experiences between age 0-16 |  |  |  |  |  |  |
| 0 | 4.6 (3.4-6.1) | 11.1 (9.2-13.5) | 5.5 (4.6) | 10.7 (7.8-14.4) | 21.7 (17.4-26.8) | 6.9 (5.7) |
| 1 | 4.2 (2.9-6.2) | 12.0 (9.6-15.0) | 6.0 (4.6) | 6.3 (3.7-10.2) | 26.9 (21.6-33.0) | 7.5 (6.0) |
| ≥ 2 | 14.9 (11.5-18-9) | 20.9 (17.0-25.3) | 7.4 (5.2) | 19.7 (14.3-26.2) | 31.5 (25.0-39.0) | 8.8 (6.7) |
| Carer status |  |  |  |  |  |  |
| Carer | 3.1 (0.4-19.1) | 22.2 (10.3-41.4) | 7.9 (5.8) | 0 (0-0) | 38.1 (20.3-59.8) | 8.7 (6.7) |
| Non-carer | 6.2 (5.0-7.7) | 13.2 (11.4-15.3) | 5.9 (4.8) | 12.3 (9.3-16.0) | 26.6 (22.2-31.4) | 7.7 (6.4) |

^a^Index of Multiple Deprivation, ^b^Short Mood and Feelings Questionnaire

Online resource 5: Univariate associations between confounders and exposure and outcomes

| **Correlates** | **University attendance**  **OR (95% CI, p-value)** | **Any health service use**  **OR (95% CI, p-value)** | **General Practice service use**  **OR (95% CI, p-value)** | **Counselling service use**  **OR (95% CI, p-value)** | **Mental health service use**  **OR (95% CI, p-value)** | **Medication use**  **OR (95% CI, p-value)** |
| --- | --- | --- | --- | --- | --- | --- |
| Sex assigned at birth |  |  |  |  |  |  |
| Being male at birth | Reference | Reference | Reference | Reference | Reference | Reference |
| Being female at birth | 1.0 (0.87;1.15, p=0.99) | 1.51 (1.17;1.92, p=0.001) | 1.62 (1.25;2.11, p<0.001) | 1.54 (1.12;2.10, p=0.007) | 1.42 (1.01;2.01, p=0.04) | 2.01 (1.43;2.82, p<0.001) |
| Ethnicity |  |  |  |  |  |  |
| White | Reference | Reference | Reference | Reference | Reference | Reference |
| Minoritized | 1.01 (0.70;1.45, p=0.96) | 0.98 (0.52;1.84, p=0.95) | 0.81 (0.46;1.83, p=0.81) | 0.90 (0.39;2.07, p=0.81) | 1.29 (0.59;2.81, p=0.53) | 0.62 (0.23;1.68, p=0.35) |
| Sexual orientation |  |  |  |  |  |  |
| Heterosexual | Reference | Reference | Reference | Reference | Reference | Reference |
| Minoritized | 1.28 (1.05;1.55, p=0.01) | 3.19 (2.40;4.23, p<0.001) | 3.39 (2.51;4.57, p<0.001) | 3.04 (2.12;4.35, p<0.001) | 3.29 (2.25;4.81, p<0.001) | 3.44 (2.37;4.98, p<0.001) |
| Maternal highest education |  |  |  |  |  |  |
| Compulsory only | Reference | Reference | Reference | Reference | Reference | Reference |
| Above compulsory | 5.55 (4.27;7.22, p<0.001) | 0.94 (0.69;1.27, p=0.68) | 0.93 (0.67;1.29, p=0.676) | 1.11 (0.76;1.61, p=0.59) | 2.10 (1.45;3.05, p<0.001) | 0.91 (0.61;1.37, p=0.65) |
| Family composition at gestation |  |  |  |  |  |  |
| Married or cohabiting | Reference | Reference | Reference | Reference | Reference | Reference |
| Not married or co-habiting | 0.54 (0.45;0.64, p<0.001) | 2.10 (1.57;2.73, p<0.001) | 1.98 (1.48;2.66, p<0.001) | 2.11 (1.49;2.97, p<0.001) | 1.13 (0.76;1.70, p=0.53) |  |
| IMD quintile at gestation |  |  |  |  |  |  |
| Least deprived | Reference | Reference | Reference | Reference | Reference | Reference |
| 2^nd^ least deprived | 0.69 (0.50;0.89, p=0.006) | 0.81 (0.49;1.34, p=0.41) | 0.69 (0.38;1.24, p=0.22) | 0.61 (0.29;1.26, p=0.18) | 0.66 (0.30;1.43, p=0.29) | 0.96 (0.50;1.85, p=0.90) |
| 3^rd^ least deprived | 0.54 (0.40;0.71, p<0.001) | 1.03 (0.63;1.69, p=0.89) | 1.19 (0.71;2.01, p=0.51) | 1.25 (0.69;2.27, p=0.46) | 1.16 (0.60;2.27, p=0.66) | 1.32 (0.71;2.45, p=0.39) |
| 4^th^ least deprived | 0.42 (0.32;0.55, p<0.001) | 0.95 (0.58;1.57, p=0.85) | 1.19 (0.71;2.01, p=0.50) | 1.04 (0.55;1.95, p=0.91) | 0.82 (0.39;1.73, p=0.61) | 1.08 (0.56;2.08, p=0.83) |
| Most deprived | 0.28 (0.22;0.37, p<0.001) | 1.67 (1.10;2.57, p=0.02) | 1.93 (1.21;3.05, p=0.005) | 1.43 (0.81;2.55, p=0.22) | 1.48 (0.79;2.74, p=0.22) | 1.77 (0.99;3.13, p=0.05) |
| Autistic traits at age 16 |  |  |  |  |  |  |
| Below threshold | Reference | Reference | Reference | Reference | Reference | Reference |
| Above threshold | 0.47 (0.35;0.63, p<0.001) | 2.55 (1.70;3.82, p<0.001) | 2.69 (1.76;4.10, p<0.001) | 2.61 (1.60;4.28, p<0.001) | 3.68 (2.28;5.94, p<0.001) | 2.50 (1.51;4.13, p<0.001) |
| sMFQ score at age 17 |  |  |  |  |  |  |
| < 12 | Reference | Reference | Reference | Reference | Reference | Reference |
| ≥ 12 | 0.45 (0.36;0.55, p <0.001) | 3.37 (2.48;4.57, p<0.001) | 3.40 (2.47;4.68, p<0.001) | 3.02 (2.05;4.45, p<0.001) | 3.34 (2.21;5.03, p<0.001) | 3.98 (2.71;5.85, p<0.001) |
| Disability at age 18 |  |  |  |  |  |  |
| A reported disability | Reference | Reference | Reference | Reference | Reference | Reference |
| No reported disability | 1.11 (0.86;1.43, p=0.41) | 0.37 (0.26;0.55, p<0.001) | 0.37 (0.25;0.54, p<0.001) | 0.34 (0.22;0.53, p<0.001) | 0.34 (0.21;0.55, p<0.001) | 0.33 (0.21;0.52, p<0.001) |
| Carer status at age 22 |  |  |  |  |  |  |
| Carer | Reference | Reference | Reference | Reference | Reference | Reference |
| Not at carer | 2.11 (1.31;3.40, p=0.002) | 1.39 (0.50;3.85, p=0.53) | 1.64 (0.51; 5.28, p=0.41) | 3.52 (0.48;25.58, p=0.21 | 0.92 (0.28; 2.98, p=0.88) | 3.15 (0.43; 22.98, p=0.26) |
| Adverse childhood experiences between age 0-16 |  |  |  |  |  |  |
| 0 | Reference | Reference | Reference | Reference | Reference | Reference |
| 1 | 1.04 (0.90;1.21, p=0.59) | 0.84 (0.63;1.12, p=0.25) | 0.86 (0.63;1.17, p=0.36) | 0.82 (0.56;1.21, p=0.33) | 0.83 (0.54;1.28, p=0.41) | 0.82 (0.55;1.21, p=0.30) |
| ≥ 2 | 0.87 (0.73;1.21, p=0.11) | 2.39 (1.85;3.10, p<0.001) | 1.98 (1.48;2.66, p<0.001) | 2.72 (1.97;3.74, p<0.001) | 2.74 (1.92;3.89, p<0.001) | 2.49 (1.79;3.46, p<0.001) |

**Online Resource 6: Associations with any Health Service use for a mental health problem**

| **Correlates** | **Model 1 n=2,649**  **OR (95% CI)** | **Model 2 n=1,949**  **aOR (95% CI)** | **Model 3 n=1,935**  **aOR (95% CI)** | **Model 4 n=991**  **aOR (95% CI)** | **Model 5 n=503**  **aOR (95% CI)** |
| --- | --- | --- | --- | --- | --- |
| Attended university |  |  |  |  |  |
| Did not attend university | Reference | Reference | Reference | Reference | Reference |
| Attended university | 0.54 (0.40;0.73, p<0.001) | 0.44 (0.30;0.63, p<0.001) | 0.47 (0.32;0.68, p<0.001) | 0.45 (0.25;0.79, p=0.006) | 0.38 (0.15;0.94, p=0.04) |
| Sex assigned at birth |  |  |  |  |  |
| Male |  | Reference | Reference | Reference | Reference |
| Female |  | 1.55 (1.01;2.39, p=0.05) | 1.50 (0.98;2.30, p=0.07) | 1.64 (0.88;3.06, p=0.12) | 1.11 (0.46;2.69, p=0.81) |
| Ethnicity |  |  |  |  |  |
| White |  | Reference | Reference | Reference | Reference |
| Minoritized |  | 0.68 (0.24;1.93, p=0.47) | 0.65 (0.23;1.87, p=0.42) | 1 (omitted) | 1 (omitted) |
| Sexual orientation |  |  |  |  |  |
| Heterosexual |  | Reference | Reference | Reference | Reference |
| Minoritized |  | 3.23 (2.25;4.66, p<0.001) | 3.06 (2.11;4.43, p<0.001) | 4.12 (2.40;7.06, p<0.001) | 0.51 (0.11;2.38, p=0.40) |
| Family composition at gestation |  |  |  |  |  |
| Married or cohabiting |  |  | Reference | Reference | Reference |
| Not married or co-habiting |  |  | 1.42 (0.90;2.27, p=0.13) | 0.81 (0.38;1.53, p=0.42) | 0.51 (0.11;2.39, p=0.40) |
| Maternal highest education |  |  |  |  |  |
| Compulsory only |  |  |  | Reference | Reference |
| Above compulsory |  |  |  | 0.74 (0.36;1.53, p=0.42) | 0.79 (0.29;2.12; p=0.64) |
| IMD quintile at gestation |  |  |  |  |  |
| Least deprived |  |  |  | Reference | Reference |
| 2^nd^ |  |  |  | 0.52 (0.21;1.29, p=0.16) | 0.49 (0.13;1.82; p=0.29) |
| 3^rd^ |  |  |  | 1.31 (0.64;2.70, p=0.45) | 1.03 (0.36;2.96; p=0.96) |
| 4^th^ |  |  |  | 0.96 (0.43;2.11, p=0.91) | 0.98 (0.30;3.15; p=0.97) |
| Most deprived |  |  |  | 1.07 (0.47;2.44, p=0.87) | 1 (omitted) |
| Autistic traits at age 16 |  |  |  |  |  |
| Below threshold |  |  |  |  | Reference |
| Above threshold |  |  |  |  | 4.44 (1.34;14.72; p=0.02) |
| sMFQ score at age 17 |  |  |  |  |  |
| sMFQ score < 12 |  |  |  |  | Reference |
| sMFQ score ≥ 12 |  |  |  |  | 3.28 (1.30;8.29; p=0.01) |
| Disability at age 18 |  |  |  |  |  |
| A reported disability |  |  |  |  | Reference |
| No reported disability |  |  |  |  | 0.42 (0.16;1.11; p=0.08) |

**Online Resource 7: Associations with General Practice use for a mental health problem**

| **Correlates** | **Model 1 n=2,649**  **OR (95% CI)** | **Model 2 n=1,949**  **aOR (95% CI)** | **Model 3 n=1,935**  **aOR (95% CI)** | **Model 4 n=991**  **aOR (95% CI)** | **Model 5 n=454**  **aOR (95% CI)** |
| --- | --- | --- | --- | --- | --- |
| Attended university |  |  |  |  |  |
| Did not attend university | Reference | Reference | Reference | Reference | Reference |
| Attended university | 0.49 (0.36;067, p<0.001) | 0.38 (0.26;0.57, p<0.001) | 0.42 (0.28;0.62, p<0.001) | 0.39 (0.21;0.71, p=0.002) | 0.28 (0.11;0.74, p=0.01) |
| Sex assigned at birth |  |  |  |  |  |
| Male |  | Reference | Reference | Reference | Reference |
| Female |  | 1.46 (0.93;2.30, p=0.10) | 1.40 (0.89;2.21, p=0.15) | 1.70 (0.87;3.32, p=0.12) | 1.25 (0.46;3.36, p=0.66) |
| Ethnicity |  |  |  |  |  |
| White |  | Reference | Reference | Reference | Reference |
| Minoritized |  | 0.79 (0.28;2.28, p=0.67) | 0.73 (0.25;2.13, p=0.57) | 1 (omitted) | 1 (omitted) |
| Sexual orientation |  |  |  |  |  |
| Heterosexual |  | Reference | Reference | Reference | Reference |
| Minoritized |  | 3.38 (2.28;4.98, p<0.001) | 3.15 (2.12;4.67, p<0.001) | 4.24 (2.39;7.49; p<0.001) | 2.44 (0.92;6.50, p=0.07) |
| Family composition at gestation |  |  |  |  |  |
| Married or cohabiting |  |  | Reference | Reference | Reference |
| Not married or co-habiting |  |  | 1.62 (1.01;2.61, p=0.05) | 0.77 (0.34;1.76, p=0.54) | 0.66 (0.14;3.17, p=0.60) |
| Maternal highest education |  |  |  |  |  |
| Compulsory only |  |  |  | Reference | Reference |
| Above compulsory |  |  |  | 0.94 (0.45;1.97, p=0.87) | 1.25 (0.43;3.61; p=0.68) |
| IMD quintile at gestation |  |  |  |  |  |
| Least deprived |  |  |  | Reference | Reference |
| 2^nd^ |  |  |  | 0.61 (0.23;1.63, p=0.32) | 0.50 (0.11;2.25; p=0.37) |
| 3^rd^ |  |  |  | 1.68 (0.78;3.65, p=0.19) | 1.56 (0.50;4.85; p=0.44) |
| 4^th^ |  |  |  | 1.32 (0.57;3.04, p=0.51) | 1.42 (0.41;4.91; p=0.58) |
| Most deprived |  |  |  | 1.47 (0.62;3.47, p=0.39) | 1 (omitted) |
| Autistic traits at age 16 |  |  |  |  |  |
| Below threshold |  |  |  |  | Reference |
| Above threshold |  |  |  |  | 3.28 (1.19;9.03; p=0.02) |
| sMFQ score at age 17 |  |  |  |  |  |
| sMFQ score < 12 |  |  |  |  | Reference |
| sMFQ score ≥ 12 |  |  |  |  | 2.47 (0.55;10.97; p=0.24) |
| Disability at age 18 |  |  |  |  |  |
| A reported disability |  |  |  |  | Reference |
| No reported disability |  |  |  |  | 0.55 (0.18;1.62; p=0.28) |

**Online Resource 8: Associations with Counselling use for a mental health problem**

| **Correlates** | **Model 1 n=2,649**  **OR (95% CI)** | **Model 2 n=1,949**  **aOR (95% CI)** | **Model 3 n=1,935**  **aOR (95% CI)** | **Model 4 n=991**  **aOR (95% CI)** | **Model 5 n=454**  **aOR (95% CI)** |
| --- | --- | --- | --- | --- | --- |
| Attended university |  |  |  |  |  |
| Did not attend university | Reference | Reference | Reference | Reference | Reference |
| Attended university | 0.58 (0.40;0.84, p=0.004) | 0.49 (0.31;0.78, p=0.003) | 0.51 (0.32;0.83, p=0.006) | 0.38 (0.18;0.78, p=0.008) | 0.28 (095;0.83, p=0.02) |
| Sex assigned at birth |  |  |  |  |  |
| Male |  | Reference | Reference | Reference | Reference |
| Female |  | 1.43 (0.84;2.47, p=0.10) | 1.41 (0.82;2.42, p=0.22) | 1.69 (0.75;3.80, p=0.20) | 1.09 (0.36;3.29, p=0.88) |
| Ethnicity |  |  |  |  |  |
| White |  | Reference | Reference | Reference | Reference |
| Minoritized |  | 0.61 (0.14;2.58, p=0.50) | 0.62 (0.15;2.63, p=0.52) | 1 (omitted) | 1 (omitted) |
| Sexual orientation |  |  |  |  |  |
| Heterosexual |  | Reference | Reference | Reference | Reference |
| Minoritized |  | 2.38 (1.49;3.82, p<0.001) | 2.30 (1.42;3.70, p=0.001) | 3.12 (1.54;6.29, p=0.001) | 2.45 (0.81;7.44, p=0.11) |
| Family composition at gestation |  |  |  |  |  |
| Married or cohabiting |  |  | Reference | Reference | Reference |
| Not married or co-habiting |  |  | 1.13 (0.60;2.10, p=0.71) | 0.47 (0.14;1.63, p=0.26) | 0.36 (0.04;3.05, p=0.34) |
| Maternal highest education |  |  |  |  |  |
| Compulsory only |  |  |  | Reference | Reference |
| Above compulsory |  |  |  | 0.83 (0.34;2.17, p=0.75) | 0.84 (0.24;2.92; p=0.78) |
| IMD quintile at gestation |  |  |  |  |  |
| Least deprived |  |  |  | Reference | Reference |
| 2^nd^ |  |  |  | 0.70 (0.23;2.08, p=0.52) | 0.38 (0.07;2.20; p=0.28) |
| 3^rd^ |  |  |  | 1.43 (0.57;3.57, p=0.45) | 1.21 (0.34;4.33; p=0.77) |
| 4^th^ |  |  |  | 1.30 (0.49;3.38, p=0.60) | 1.31 (0.33;5.14; p=0.70) |
| Most deprived |  |  |  | 0.59 (0.16;2.21, p=0.44) | 1 (omitted) |
| Autistic traits at age 16 |  |  |  |  |  |
| Below threshold |  |  |  |  | Reference |
| Above threshold |  |  |  |  | 3.15 (0.99;9.96; p=0.05) |
| sMFQ score at age 17 |  |  |  |  |  |
| sMFQ score < 12 |  |  |  |  | Reference |
| sMFQ score ≥ 12 |  |  |  |  | 3.57 (0.74;17.14; p=0.11) |
| Disability at age 18 |  |  |  |  |  |
| A reported disability |  |  |  |  | Reference |
| No reported disability |  |  |  |  | 0.35 (0.11;1.09; p=0.07) |

**Online Resource 9: Associations with Mental Health Service use for a mental health problem**

| **Correlates** | **Model 1 n=2,649**  **OR (95% CI)** | **Model 2 n=1,949**  **aOR (95% CI)** | **Model 3 n=1,935**  **aOR (95% CI)** | **Model 4 n=991**  **aOR (95% CI)** | **Model 5 n=454**  **aOR (95% CI)** |
| --- | --- | --- | --- | --- | --- |
| Attended university |  |  |  |  |  |
| Did not attend university | Reference | Reference | Reference | Reference | Reference |
| Attended university | 0.66 (0.43;1.01, p=0.06) | 0.66 (0.39;1.13, p=0.13) | 0.69 (0.41;1.17, p=0.17) | 0.60 (0.27;1.37, p=0.23) | 0.37 (0.10;1.42, p=0.15) |
| Sex assigned at birth |  |  |  |  |  |
| Male |  | Reference | Reference | Reference | Reference |
| Female |  | 1.35 (0.76;2.39, p=0.31) | 1.32 (0.74;2.35, p=0.35) | 1.55 (0.65;3.69, p=0.32) | 1.27 (0.34;4.75, p=0.72) |
| Ethnicity |  |  |  |  |  |
| White |  | Reference | Reference | Reference | Reference |
| Minoritized |  | 0.72 (0.17;3.00, p=0.64) | 0.65 (0.15;2.79, p=0.57) | 1 (omitted) | 1 (omitted) |
| Sexual orientation |  |  |  |  |  |
| Heterosexual |  | Reference | Reference | Reference | Reference |
| Minoritized |  | 2.88 (1.75;4.74, p<0.001) | 2.83 (1.72;4.67, p<0.001) | 3.33 (1.59;6.97, p=0.001) | 0.67 (0.14;3.18, p=0.63) |
| Family composition at gestation |  |  |  |  |  |
| Married or cohabiting |  |  | Reference | Reference | Reference |
| Not married or co-habiting |  |  | 1.51 (0.82;2.81, p=0.19) | 0.97 (0.35;2.69, p=0.96) | 0.87 (0.09;8.08, p=0.90) |
| Maternal highest education |  |  |  |  |  |
| Compulsory only |  |  |  | Reference | Reference |
| Above compulsory |  |  |  | 1.12 (0.32;2.75, p=0.80) | 1.32 (0.35;5.04; p=0.68) |
| IMD quintile at gestation |  |  |  |  |  |
| Least deprived |  |  |  | Reference | Reference |
| 2^nd^ |  |  |  | 0.33 (0.07;1.55, p=0.16) | 0.68 (0.11;4.12; p=0.68) |
| 3^rd^ |  |  |  | 1.49 (0.57;3.90, p=0.42) | 1.24 (0.30;5.11; p=0.77) |
| 4^th^ |  |  |  | 0.97 (0.32;2.97, p=0.96) | 0.33 (0.03;3.38; p=0.35) |
| Most deprived |  |  |  | 1.40 (0.48;4.05, p=0.54) | 1 (omitted) |
| Autistic traits at age 16 |  |  |  |  |  |
| Below threshold |  |  |  |  | Reference |
| Above threshold |  |  |  |  | 5.14 (1.07;24.53; p=0.04) |
| sMFQ score at age 17 |  |  |  |  |  |
| sMFQ score < 12 |  |  |  |  | Reference |
| sMFQ score ≥ 12 |  |  |  |  | 5.47 (1.48;20.19; p=0.01) |
| Disability at age 18 |  |  |  |  |  |
| A reported disability |  |  |  |  | Reference |
| No reported disability |  |  |  |  | 0.29 (0.08;1.05; p=0.06) |

**Online Resource 10: Associations with Medication use for a mental health problem**

| **Correlates** | **Model 1 n=2,649**  **OR (95% CI)** | **Model 2 n=1,949**  **aOR (95% CI)** | **Model 3 n=1,935**  **aOR (95% CI)** | **Model 4 n=991**  **aOR (95% CI)** | **Model 5 n=653**  **aOR (95% CI)** |
| --- | --- | --- | --- | --- | --- |
| Attended university |  |  |  |  |  |
| Did not attend university | Reference | Reference | Reference | Reference | Reference |
| Attended university | 0.49 (0.34;0.72, p<0.001) | 0.42 (0.26;0.66, p<0.001) | 0.45 (0.28;0.72, p=0.001) | 0.35 (0.17;0.72, p=0.005) | 0.20 (0.06;0.60, p=0.004) |
| Sex assigned at birth |  |  |  |  |  |
| Male |  | Reference | Reference | Reference | Reference |
| Female |  | 2.16 (1.17;3.97, p=0.01) | 2.09 (1.13;3.84, p=0.02) | 1.94 (0.83;4.57, p=0.13) | 1.59 (0.50;5.03, p=0.43) |
| Ethnicity |  |  |  |  |  |
| White |  | Reference | Reference | Reference | Reference |
| Minoritized |  | 0.87 (2.01;5.08, p=0.82) | 0.80 (0.24;2.72, p=0.73) | 1 (omitted) | 1 (omitted) |
| Sexual orientation |  |  |  |  |  |
| Heterosexual |  | Reference | Reference | Reference | Reference |
| Minoritized |  | 3.20 (2.01;5.08, p<0.001) | 3.02 (1.89;4.83, p<0.001) | 4.18 (2.07;8.46, p<0.001) | 1.99 (0.65;6.11, p=0.23) |
| Family composition at gestation |  |  |  |  |  |
| Married or cohabiting |  |  | Reference | Reference | Reference |
| Not married or co-habiting |  |  | 1.58 (0.90;2.78, p=0.11) | 0.61 (0.20;1.85, p=0.38) | 0.41 (0.05;3.55, p=0.42) |
| Maternal highest education |  |  |  |  |  |
| Compulsory only |  |  |  | Reference | Reference |
| Above compulsory |  |  |  | 0.89 (0.35;2.25, p=0.80) | 1.51 (0.46;4.97; p=0.50) |
| IMD quintile at gestation |  |  |  |  |  |
| Least deprived |  |  |  | Reference | Reference |
| 2^nd^ |  |  |  | 0.75 (0.25;2.30, p=0.62) | 0.75 (0.17;3.22; p=0.70) |
| 3^rd^ |  |  |  | 1.77 (0.71;4.42, p=0.22) | 1.24 (0.34;5.47; p=0.74) |
| 4^th^ |  |  |  | 0.84 (0.27;2.57, p=0.76) | 0.58 (0.11;3.14; p=0.52) |
| Most deprived |  |  |  | 1.05 (0.34;3.25, p=0.93) | 1 (omitted) |
| Autistic traits at age 16 |  |  |  |  |  |
| Below threshold |  |  |  |  | Reference |
| Above threshold |  |  |  |  | 3.89 (0.84;17.88; p=0.08) |
| sMFQ score at age 17 |  |  |  |  |  |
| sMFQ score < 12 |  |  |  |  | Reference |
| sMFQ score ≥ 12 |  |  |  |  | 3.83 (1.24;11.83; p=0.02) |
| Disability at age 18 |  |  |  |  |  |
| A reported disability |  |  |  |  | Reference |
| No reported disability |  |  |  |  | 0.79 (0.21;2.99; p=0.73) |

**Online resource 11: association between university attendance and health service use for a mental health problem by age 24 only in individuals with probable depression at age 17**

| **Correlate** | **Any health service use for a mental health problem by age 24 (n=306)**  **OR (95% CI)** |
| --- | --- |
| Attended university |  |
| Did not attend university | Reference |
| Attended university | 0.71 (0.38;1.31, p=0.27) |

**Online Resource 12: Interaction graphs for predicted probability of health service use for a mental health problem by age 24 in complete case analysis**

**Figure 1: predicted probability of health service use for a mental health problem by age 24 for the interaction between university attendance status and sex**

**
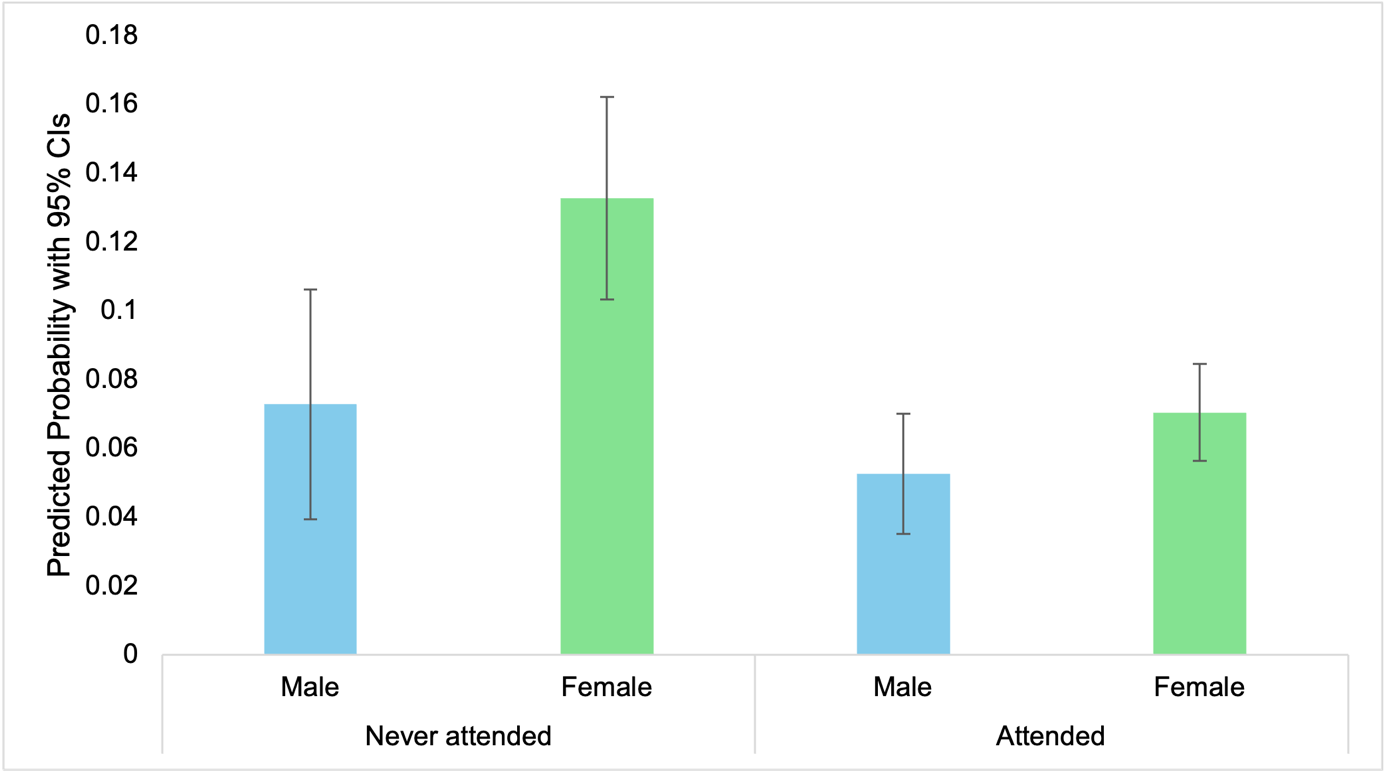
**

**Figure 2: predicted probability of health service use for a mental health problem by age 24 for the interaction between university attendance status and sex adjusting for confounders^1^**

**
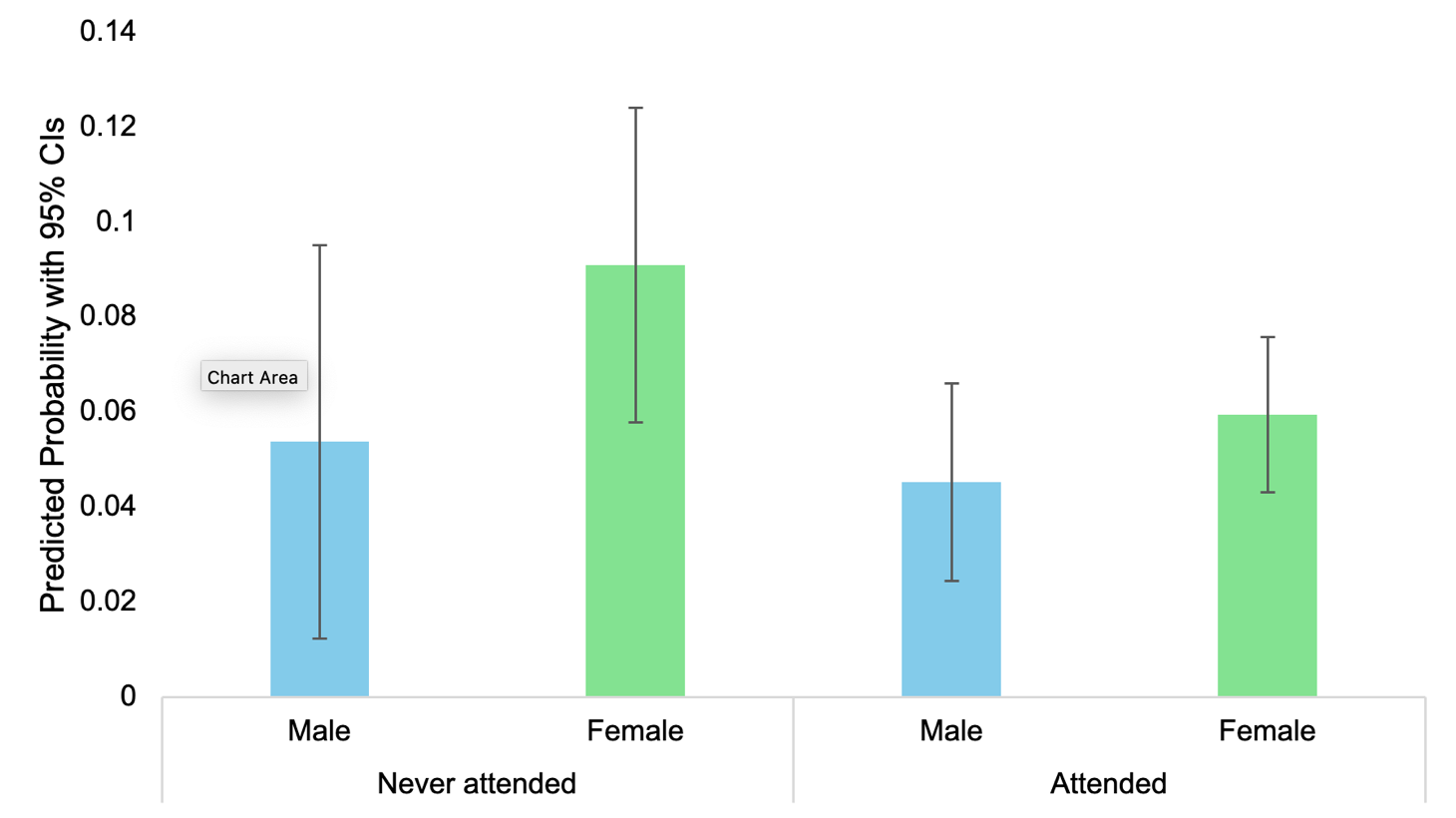
**

*Legend:1= adjusting for confounders of depression score reported at age 17, autistic traits reported at age 16.*

**Figure 3: predicted probability of health service use for a mental health problem by age 24 for the interaction between university attendance status and ethnicity**

**
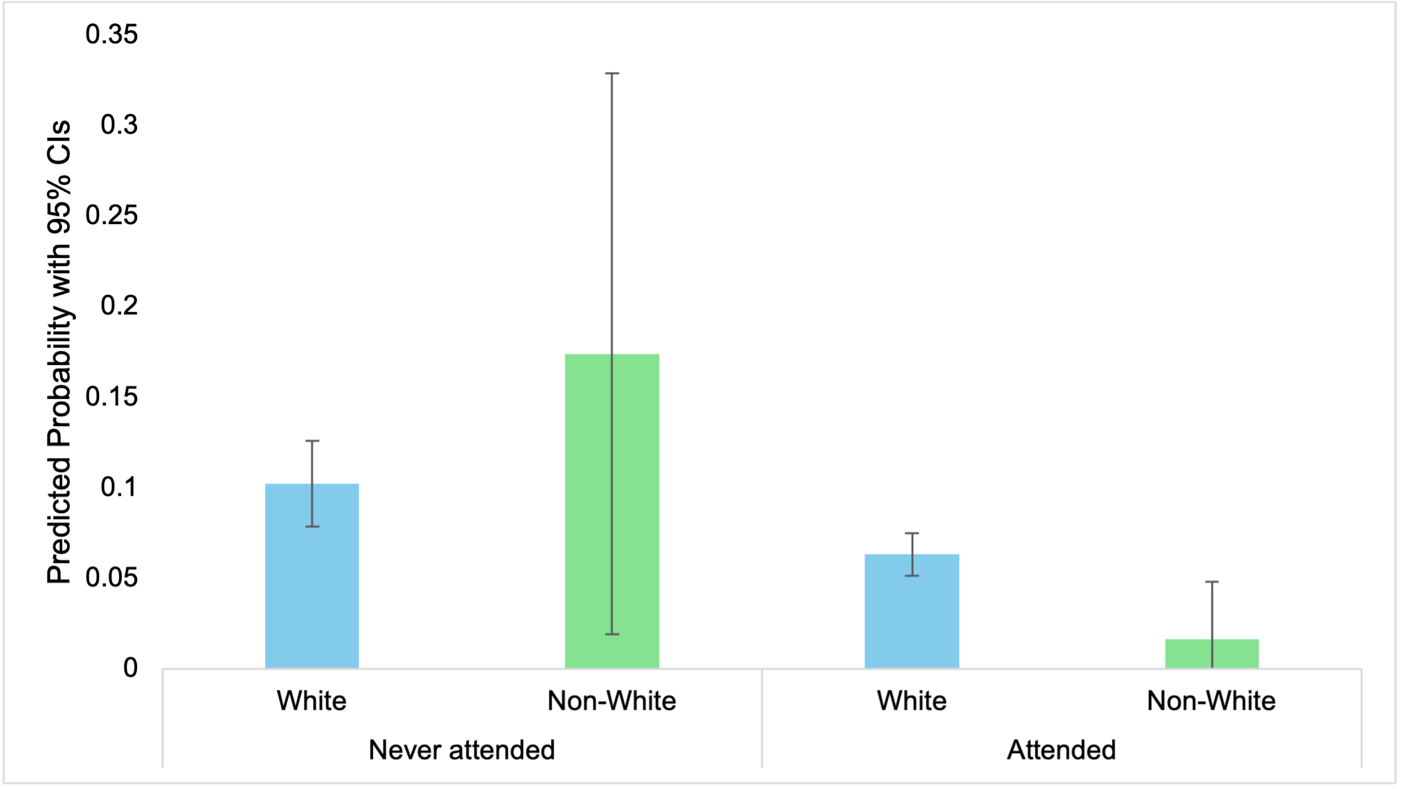
**

**Figure 4: predicted probability of health service use for a mental health problem by age 24 for the interaction between university attendance status and sexual orientation**

**
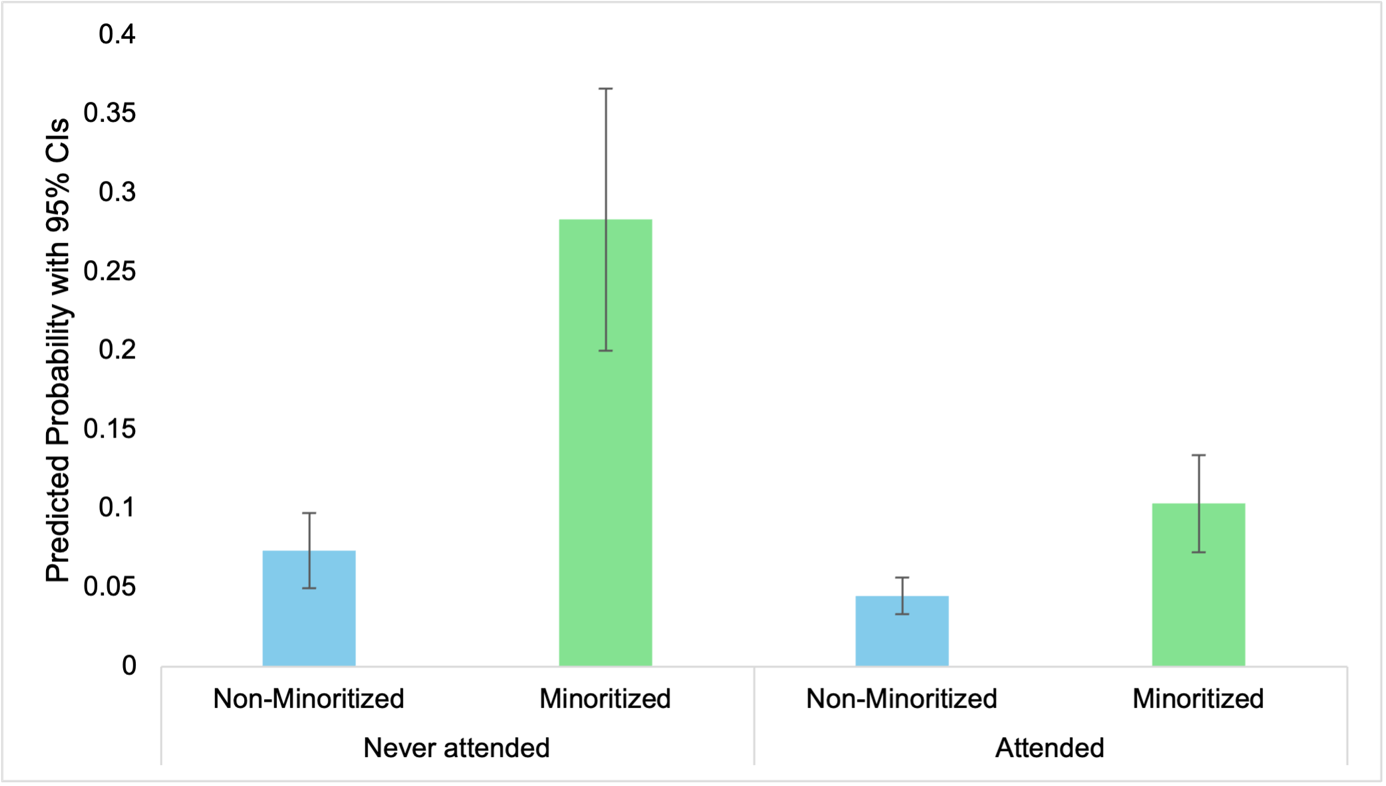
**

**Figure 5: predicted probability of health service use for a mental health problem by age 24 for the interaction between university attendance status and sexual orientation adjusting for confounders^1^**

**
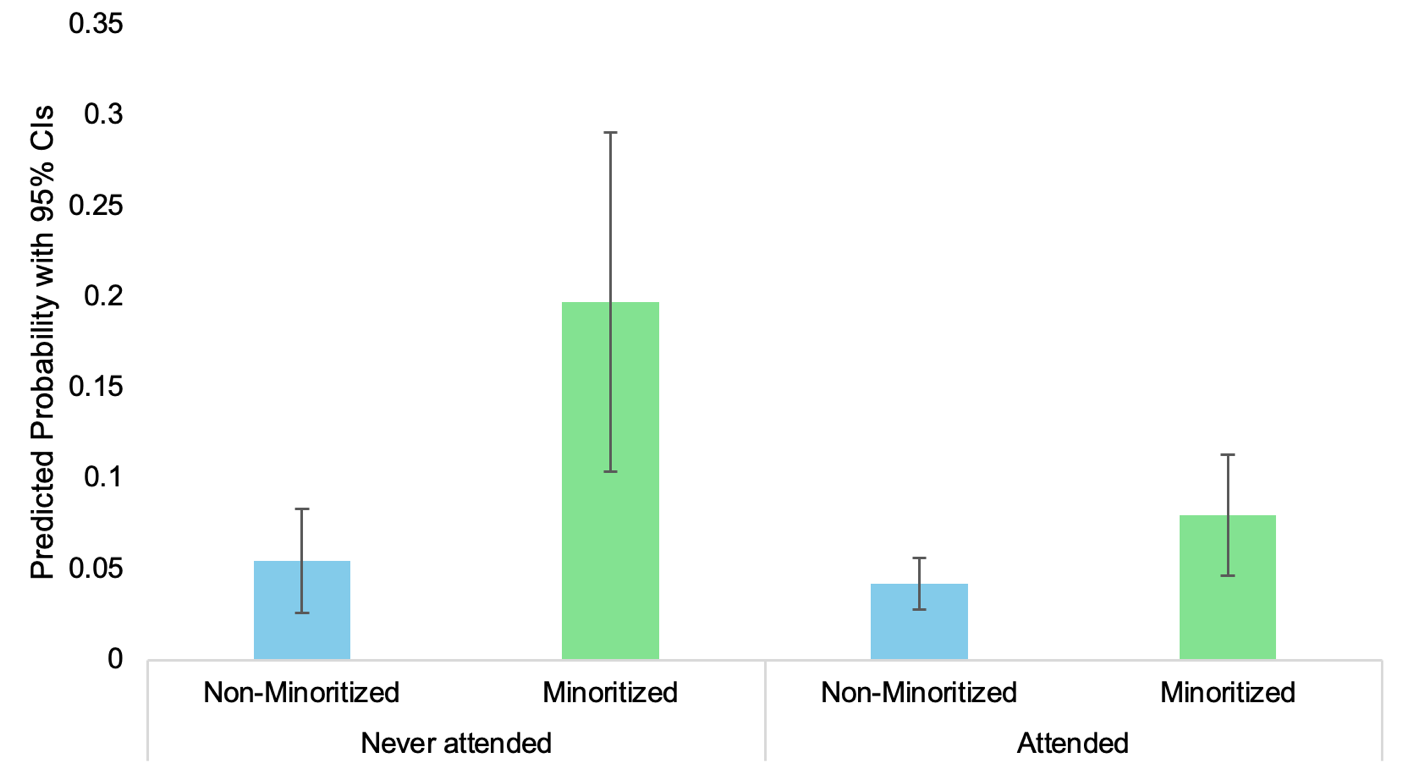
**

*Legend:1= adjusting for confounders of depression score reported at age 17, autistic traits reported at age 16.*

**Figure 6: predicted probability of health service use for a mental health problem by age 24 for the interaction between university attendance status and maternal education**

**
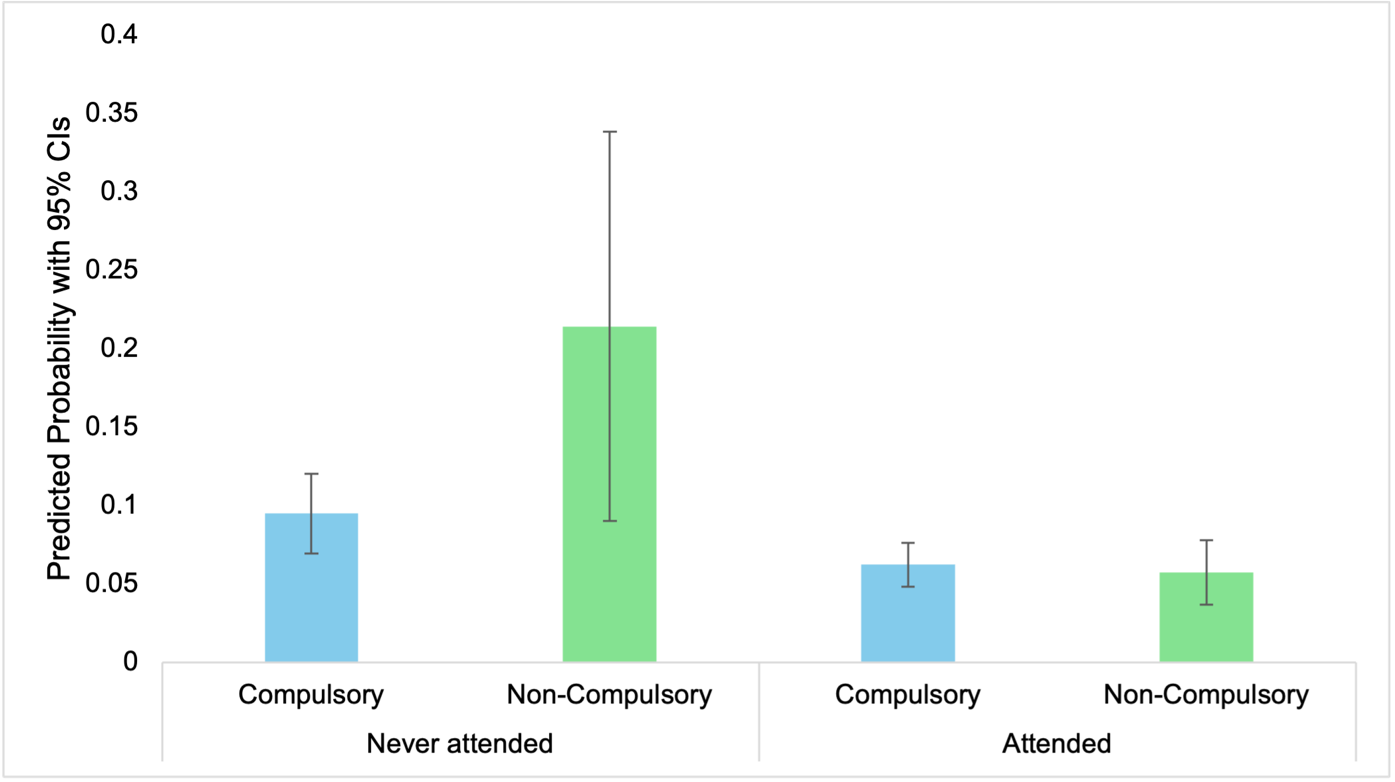
**

**Figure 7: predicted probability of health service use for a mental health problem by age 24 for the interaction between university attendance status and maternal education adjusting for confounders^1^**

**
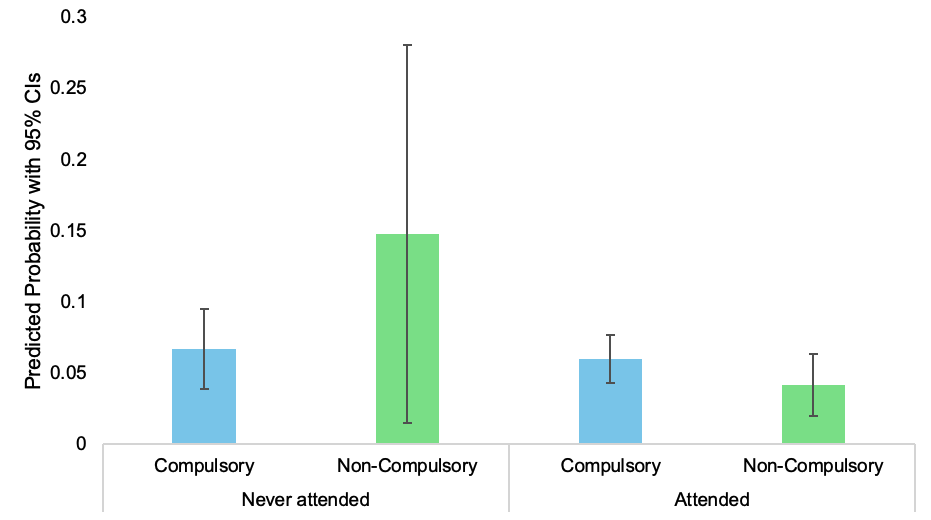
**

*Legend:1= adjusting for confounders of depression score reported at age 17, autistic traits reported at age 16.*

**References**

1. Stringaris, A., Lewis, G. & Maughan, B. 2014. Developmental pathways from childhood conduct problems to early adult depression: findings from the ALSPAC cohort. *The British Journal of* *Psychiatry,* 205, 17-23.
2. Hammerton, G., Lewis, G., Heron, J., Fernandes, G., Hickman, M. & Lewis, G. 2023. The association of alcohol dependence and consumption during adolescence with depression in young adulthood, in England: a prospective cohort study. *The Lancet Psychiatry*.
3. Reiss, F., Meyrose, A. K., Otto, C., Lampert, T., Klasen, F. & Ravens-Sieberer, U. 2019. Socioeconomic status, stressful life situations and mental health problems in children and adolescents: Results of the German BELLA cohort-study. *PLoS One,* 14, e0213700.
4. Torvik, F. A., Eilertsen, E. M., Mcadams, T. A., Gustavson, K., Zachrisson, H. D., Brandlistuen, R., Gjerde, L. C., Havdahl, A., Stoltenberg, C., Ask, H. & Ystrom, E. 2020. Mechanisms linking parental educational attainment with child ADHD, depression, and academic problems: a study of extended families in The Norwegian Mother, Father and Child Cohort Study. *Journal of Child Psychology and* *Psychiatry,* 61, 1009-1018.
5. Erola, J., Jalonen, S. & Lehti, H. 2016. Parental education, class and income over early life course and children's achievement. *Research in Social Stratification and Mobility,* 44, 33-43.
